# Abstracts from the 5th International Conference for Healthcare and Medical Students (ICHAMS): Dublin, Ireland. 19-20 February 2016

**DOI:** 10.1186/s12919-016-0004-2

**Published:** 2016-07-05

**Authors:** Yvonne Sweeney, Hugh O’Neill, Garry Duffy, Daniel Creegan, Viviana Bustos, Brian J. Harvey, Alexander Dalphy, Anthony O’Grady, Elaine Kay, Katarzyna Samelska, Justyna Izdebska, Anna Kurowska, Rena Al-Zubaidy, Magdalena Mroz, Brian Harvey, Ryan Fagan, Helen French, Vanessa Cuddy, Jennifer Ashton, Michelle Clarke, Zaid Sayedalamin, Mukhtiar Baig, Osama Almutairi, Hassan Allam, Taher F. Halawa, Hazem M. Atta, Linda Sirone, Renars Erts, Jana Pavare, Louis Richter, Joseph Morris, Irene Oglesby, Eimear Dunne, Dermot Kenny, Ibrahim Mohammed Mahdi Khayyat, Viviana Bustos Salgado, Brian J. Harvey, Jonavan Tan, Daragh Moneley, Austin Leahy, Patricia Fitzgerald, Evelyn Fennelly, Grace Harkin, John Lee, Erica O’Sullivan, Brian Kirby, Sarah O’Neill, Diana Gonciar, Teodora Mocan, Cristian Matea, Lucian Mocan, Cornel Iancu, Chloe Doran, Sarah Hoolahan, Tobias Engel, Maha Alkhattab, Tijna Alekseeva, William Lackington, Fergal O’Brien, Nabeehah Moollan, Chloe Doran, Noel Gerry McElvaney, Cedric Gunaratnam, Lorraine Scanlon, Noeleen Brady, Suzanne Timmons, Richard Bresler, Zita Abreu, Stefan Trohonel, Joanne Bargman, Ahmad A. Mirza, Ahmed Badrek-Amoudi, Rakan H. Aun, Hussam A. Senan, Abdulrahim A. Mirza, Mohammed S. Binsaad, Mian U. Farooq, Saheli Nandi, Beatrice D’Orsi, Jochen Prehn, Mariia Zharova, Pavel Umrukhin, Wendy Evans-Uhegbu, Frank Doyle, Hope Kudryashova, Derek Dorris, Anthony Cummins, Jemma Doheny-Shanley, Mark Woodward, Wesley Hayes, Marina Yostos, David Cotter, Melanie Focking, Samantha Stancu, Florin Iordache, Bogdan A. Popescu, Muhammad-Mujtaba A. Akanmu, Alero A. Robert, Ezekiel O. Oridota, Ahmad A. Mirza, Ali K. Alzahrani, Omar Alfarhan, Essam Nour Eldin, Bronagh MacManus, Owen Keane, Patrick Hillery, James Lee, Hugh O’Reilly, Niamh Collins, Ibrahim Abu saq, Abdullah Al Mufarrih, Muath Jaafari, Abdullah Al Mahayni, Amen Bawazir, Sultan Alkhateeb, Amenah Dhannoon, Damir Vareslija, Arnold Hill, Leonie Young, Declan Donoghue, Criona Walsh, Aileen McCabe, John Pope, Saturnino Pasco, Caroline Fallon, Don Solanki, Fiona Kiernan, Sinead Galvin, Jquan Mucvimicc, Johanna Mulvihill, Ahmad A. Mirza, Soha A. Elmosry, Lorenza Andres Ameida De Souza, Yuri de Oliveira, Diego Menezes, Alene Vanessa Santos, Ahmad Zaki Asraf, Raymond Stallings, Olga Piskareva, Ross Conlon, Siún Sweeney-Landers, Cathy Burke, Paraic Behan, Seamus Sreenan, Ahmed Organjee, Tatsiana Crosbie-Staunton, Emer Reeves, Noel McElvaney, Crystal Mieres, Leonie Young, Sara Charmsaz, Aya Al-Jalamdeh, Mary Corcoran, Martha McElligott, Niall Stevens, Hilary Humphreys, Rashid Barnawi, Abdulaziz Ghurab, Sultan Alfaer, Hassan Balubaid, Kamal Hanbazazah, Mohammed Bukhari, Mohammed Alsakkaf, Ahmad Mirza, Amrallah Mohammed, Anastasia Pratanata, Maria Nathania, Tsabita Annisa, Beti Dewi, Kuok Zhen Lee, Tomas P. Carroll, Laura Fee, Noel G. McElvaney, Rachel C. White, Robert T Brady, Fergal O’Brien

**Affiliations:** Royal College of Surgeons in Ireland, Dublin, Ireland; Royal College of Surgeons in Ireland, Dublin, Ireland; Department of Molecular Medicine, Royal College of Surgeons in Ireland, Beaumont Hospital, Dublin, Ireland; Royal College of Surgeons in Ireland, Dublin, Ireland; Department of Pathology, Beaumont Hospital, Royal College of Surgeons in Ireland, Dublin, Ireland; Department of Ophthalmology, Second Faculty of Medicine, Medical University of Warsaw, Warsaw, Poland; Royal College of Surgeons in Ireland, Dublin, Ireland; Department of Molecular Medicine, Beaumont Hospital, Royal College of Surgeons in Ireland, Dublin, Ireland; Department of Physiotherapy, Royal College of Surgeons in Ireland, Dublin, Ireland; Department of Physiotherapy, Beaumont Hospital, Dublin, Ireland; Rabigh Faculty of Medicine, King Abdulaziz University, Jeddah, Saudi Arabia; Department of Clinical Biochemistry, Rabigh Faculty of Medicine, King Abdulaziz University, Jeddah, Saudi Arabia; Department of Pediatrics, Rabigh Faculty of Medicine, King Abdulaziz University, Jeddah, Saudi Arabia; Riga Stradins University, Riga, Latvia; Royal College of Surgeons in Ireland, Dublin, Ireland; Molecular and Cell Therapeutics, Royal College of Surgeons in Ireland, Dublin, Ireland; Clinical Research Centre, Royal College of Surgeons in Ireland, Dublin, Ireland; Royal College of Surgeons in Ireland, Dublin, Ireland; Department of Molecular Medicine, Beaumont Hospital, Royal College of Surgeons, Dublin, Ireland; Royal College of Surgeons in Ireland, Dublin, Ireland; Department of Surgery, Beaumont Hospital, Royal College of Surgeons in Ireland, Dublin, Ireland; Non Invasive Vascular Unit, Beaumont Hospital, Royal College of Surgeons in Ireland, Dublin, Ireland; School of Medicine, National University of Ireland, Galway, Ireland; Department of Gastroenterology, University Hospital Galway, Galway, Ireland; Department of Medicine, Royal College of Surgeons in Ireland, Dublin, Ireland; School of Pharmacy, Royal College of Surgeons in Ireland, Dublin, Ireland; MCT Department, Royal College of Surgeons in Ireland, Dublin, Ireland; Department of Medicine, University of Medicine and Pharmacy Iuliu Hatieganu, Cluj-Napoca, Romania; Regional Institute of Gastroenterology and Hepatology, Cluj-Napoca, Romania; Royal College of Surgeons in Ireland, Dublin, Ireland; Physiology & Medical Physics, Royal College of Surgeons in Ireland, Dublin, Ireland; Department of Medicine, Royal College of Surgeons in Ireland, Dublin, Ireland; Department of Anatomy, Royal College of Surgeons, Dublin, Ireland; Department of Medicine, Royal College of Surgeons in Ireland, Dublin, Ireland; Beaumont Hospital, Beaumont, Dublin, Ireland; School of Medicine, University College Cork, Cork City, Ireland; Centre for Gerontology and Rehabilitation, University College Cork, St Finbarr’s Hospital, Cork City, Ireland; Department of Medicine, Royal College of Surgeons in Ireland, Dublin, Ireland; Department of Nephrology, University Hospital Network, Toronto, Canada; Home Peritoneal Dialysis Unit, University Hospital Network, Toronto, Ontario Canada; College of Medicine, Taif University, Taif, Saudi Arabia; Department of Surgery, Faculty of Medicine, Umm Al-Qura University, Makkah, Saudi Arabia; Dr. Soliman Fakeeh Hospital, Jeddah, Saudi Arabia; Department of Otolaryngology-Head and Neck Surgery, Alnoor Specialist Hospital, Makkah, Saudi Arabia; Faculty of Medicine, Umm Al-Qura University, Makkah, Saudi Arabia; Department of Strategic Planning and Institutional Advancement, King Abdullah Medical City, Makkah, Saudi Arabia; Department of Medicine, Royal College of Surgeons in Ireland, Dublin, Ireland; The Centre for the Study of Neurological Disorders, Royal College of Surgeons in Ireland, Dublin, Ireland; I.M. Sechenov First Moscow State Medical University, Moscow, 119435 Russia; Department of Medicine, Royal College of Surgeons in Ireland, Dublin, Ireland; Department of Psychology, Royal College of Surgeons in Ireland, Dublin, Ireland; School of Psychology, University College Cork, Cork, Ireland; Department of General Practice, Royal College of Surgeons in Ireland, Dublin, Ireland; Department of Medicine, University of Bristol, Bristol, UK; University Hospital Bristol NHS Foundation trust, University of Bristol, Bristol, UK; Royal College of Surgeons in Ireland, Dublin, Ireland; Department of Psychiatry, Royal College of Surgeons in Ireland, Dublin, Ireland; Carol Davila University of Medicine and Pharmacy, Bucharest, 020022 Romania; Bucharest Clinical Emergency Hospital, Bucharest, 014461 Romania; University of Lagos, Lagos, Nigeria; Faculty of Medicine, Umm Al-Qura University, Makkah, Saudi Arabia; Royal College of Surgeons in Ireland, Dublin, Ireland; Connolly Hospital Blanchardstown, Royal College of Surgeons in Ireland, Dublin, Ireland; Faculty of Medicine, King Khalid University, Abha, Saudi Arabia; Faculty of Medicine, Al Maarfe Colleges, Riyadh, Saudi Arabia; Department of Public Health, King Saud bin Abdulaziz University, Riyadh, Saudi Arabia; Department of Uritolgical Oncology, Kind Abdulaziz Medical City, Riyadh, Saudi Arabia; Royal College of Surgeons in Ireland, Dublin, Ireland; Endocrine Oncology Research Group, Department of Surgery, Royal College of Surgeons in Ireland, Dublin, Ireland; Department of Medicine, Royal College of Surgeons in Ireland, Dublin, Ireland; ICU Department, Beaumont Hospital, Beaumont, Dublin, Ireland; College of Medicine, Taif University, Taif, Saudi Arabia; Research Center, King Abdullah Medical City, Makkah, Saudi Arabia; Department of Pharmacology, Faculty of Medicine, Cairo University, Cairo, Egypt; Epidemiology and Statistics Department, King Abdullah Medical City Research Center, Makkah, Saudi Arabia; Núcleo de Biotecnologia e Bioprospecção (NBBio), Escola Bahiana de Medicina e Saúde Pública, Salvador, Brazil; Department of Medicine, Royal College of Surgeons in Ireland, Dublin, Ireland; Cancer Genetics Department, Royal College of Surgeons in Ireland, Dublin, Ireland; Molecular and Cellular Therapeutics Department, Royal College of Surgeons in Ireland, Dublin, Ireland; Department of Medicine, University College Cork, Cork, Ireland; General Gynaecology, University Maternity Hospital Cork, Cork, Ireland; Department of Medicine, Royal College of Surgeons in Ireland, Dublin, Ireland; Connolly Hospital, Connolly, Dublin, Ireland; Department of Medicine, Royal College of Surgeons in Ireland, Dublin, Ireland; Education and Research Center, Beaumont Hospital, Beaumont, Dublin, Ireland; Department of Medicine, Royal College of Surgeons in Ireland, Dublin, Ireland; Department of Surgery, Royal College of Surgeons in Ireland, Dublin, Ireland; Royal College of Surgeons in Ireland, Dublin, Ireland; Irish Pneumococcal Reference Laboratory, Temple Street Children’s University Hospital, Dublin, Ireland; Department of Clinical Microbiology, Beaumont Hospital, Royal College of Surgeons in Ireland, Dublin, Ireland; Faculty of Medicine, King Abdulaziz University, Jeddah, Saudi Arabia; Department of Medicine, Batterjee Medical College, Jeddah, Saudi Arabia; Research Center, King Abdullah Medical City, Makkah, Saudi Arabia; College of Medicine, Taif University, Taif, Saudi Arabia; Oncology Department, King Abdullah Medical City, Mekkah, Saudi Arabia; Faculty of Medicine, Universitas Indonesia, Jakarta, Indonesia; Department of Microbiology, Faculty of Medicine, Universitas Indonesia, Jakarta, Indonesia; Royal College of Surgeons in Ireland, Dublin, Ireland; Alpha One Foundation, Royal College of Surgeons in Ireland, Beaumont Hospital, Dublin, Ireland; Tissue Engineering Research Group (TERG), Royal College of Surgeons in Ireland, Dublin, Ireland

## Abstract

O1: Assessing the protective effect of dexrazoxane against doxorubicin-induced toxicity in HL-1 cardiomyocytes

Yvonne Sweeney, Hugh O’Neill, Garry Duffy

O2: Role of KCNQ1 in epithelial barrier repair

Daniel Creegan, Viviana Bustos, Brian J. Harvey

O3: The suitability of non-small cell lung cancer cytology preparations for the analysis of anaplastic lymphoma kinase gene rearrangements

Alexander Dalphy, Anthony O’Grady, Elaine Kay

O4: Penetrating keratoplasty and descemet’s stripping automated endothelial keratoplasty may lead to deterioration in glaucoma management

Katarzyna Samelska, Justyna Izdebska, Anna Kurowska

O5: The effect of Resolvin D1 on normal and cystic fibrosis human bronchial epithelium

Rena Al-Zubaidy, Magdalena Mroz, Brian Harvey

O6: Validity of clinical assessment compared with plantar fascia thickness on ultrasound for plantar fasciitis: a cross-sectional study

Ryan Fagan, Helen French, Vanessa Cuddy, Jennifer Ashton, Michelle Clarke

P1: Undergraduate medical research in Gulf Cooperation Council (GCC) countries: A descriptive study of students’ views

Zaid Sayedalamin, Mukhtiar Baig, Osama Almutairi, Hassan Allam, Taher F. Halawa, Hazem M. Atta

P2: Positive fluid balance as a prognostic factor in children with sepsis during first 3 hours of resuscitation in intensive care unit

Linda Sirone, Renars Erts, Jana Pavare

P3: Patients on aspirin: Too little or too much?

Louis Richter, Joseph Morris, Irene Oglesby, Eimear Dunne, Dermot Kenny

P4: Beta catenin/TCF4 activation reduces KCNQ1 current in colonic monolayers

Ibrahim Mohammed Mahdi Khayyat, Viviana Bustos Salgado, Brian J. Harvey

P5: Size Matters. Abdominal aortic aneurysm: adherence to surveillance imaging guidelines

Jonavan Tan, Daragh Moneley, Austin Leahy, Patricia Fitzgerald

P6: Endoscopic retrograde cholangiopancreatography in the west of Ireland: Procedural outcomes and peri-procedural complications

Evelyn Fennelly, Grace Harkin, John Lee

P7: The effect of the extracellular redox environment on polyamine-platelet interactions

Erica O’Sullivan, Brian Kirby, Sarah O’Neill

P8: Functionalized gold nanoparticles: preliminary data on in vitro toxicity and comparative photothermal effect

Diana Gonciar, Teodora Mocan, Cristian Matea, Lucian Mocan, Cornel Iancu

P9: Imaging proteasomal inhibition after seizures in the brain: A study into cellular activity in the hippocampus of epileptic transgenic mice

Chloe Doran, Sarah Hoolahan, Tobias Engel

P10: Investigating the ability of the Olfactory epithelial stem cells to differentiate into glial cells by assessing cell morphology and marker expression

Maha Alkhattab, Tijna Alekseeva, William Lackington, Fergal O’Brien

P11: Beaumont Hospital cystic fibrosis service audit and annual report

Nabeehah Moollan, Chloe Doran, Noel Gerry McElvaney, Cedric Gunaratnam

P12: Quick cognitive screening: the 6-item cognitive impairment test and the temporal orientation score

Lorraine Scanlon, Noeleen Brady, Suzanne Timmons

P13: Granular analysis of causes of peritoneal dialysis technique failure in the first six months of therapy

Richard Bresler, Zita Abreu, Stefan Trohonel, Joanne Bargman

P14: Job satisfaction of surgeons working in hajj pilgrimage: a multicenter study

Ahmad A. Mirza, Ahmed Badrek-Amoudi, Rakan H. Aun, Hussam A. Senan, Abdulrahim A. Mirza, Mohammed S. Binsaad, Mian U. Farooq

P15: Investigation of the role of Bok using wild-type, bax-, bok-, and bax/bok-double-deficient mice

Saheli Nandi, Beatrice D’Orsi, Jochen Prehn

P16: Is it possible to predict resistance of an organism to stress based on the level of corticosterone?

Mariia Zharova, Pavel Umrukhin

P17: Investigating the strength model of self-regulation (ego depletion) and medical decision making and error in medical students

Wendy Evans-Uhegbu, Frank Doyle, Hope Kudryashova, Derek Dorris, Anthony Cummins

P18: Does bladder drainage with intermittent catheterisation preserve kidney function in boys with posterior urethral valves?

Jemma Doheny-Shanley, Mark Woodward, Wesley Hayes

P19: Investigating the role of Stonin 2, a Clathrin Mediated Endocytosis adaptor protein, in altered hippocampal synaptic transmission characterized in schizophrenia

Marina Yostos, David Cotter, Melanie Focking

P20: Predicting complications after colon resection

Samantha Stancu, Florin Iordache, Bogdan A. Popescu

P21: Knowledge, attitude and practice of the methods of primary and secondary prevention of cervical cancer among NYSC members in Lagos state, Nigeria

Muhammad-Mujtaba A. Akanmu, Alero A. Robert, Ezekiel O. Oridota

P22: Incidental glucose and lipid metabolisms disorders among office workers: a cross sectional study

Ahmad A. Mirza, Ali K. Alzahrani, Omar Alfarhan, Essam Nour Eldin

P23: Differentiating clinically significant spinal injuries; a review of emergency department presentations

Bronagh MacManus, Owen Keane, Patrick Hillery, James Lee, Hugh O’Reilly, Niamh Collins

P24: Pattern of renal colic occurrence due to urinary stones during Ramadan and other months of the year at King Abdulaziz Medical City, Riyadh, KSA

Ibrahim Abu saq, Abdullah Al Mufarrih, Muath Jaafari, Abdullah Al Mahayni, Amen Bawazir, Sultan Alkhateeb

P25: Proteomic analysis reveals novel AIB1 co-factors that may contribute to acquired endocrine resistance in breast cancer

Amenah Dhannoon, Damir Vareslija, Arnold Hill, Leonie Young

P26: Improving sedation practice in general ICU in Beaumont Hospital

Declan Donoghue, Criona Walsh, Aileen McCabe, John Pope, Saturnino Pasco, Caroline Fallon, Don Solanki, Fiona Kiernan, Sinead Galvin, Jquan Mucvimicc, Johanna Mulvihill

P27: Diagnosis and control of hypertension as indicators of the level of awareness among relatives of medical students

Ahmad A. Mirza, Soha A. Elmosry

P28: Evaluation of the antitumor potential from extracts of endemic plants of Brazilian caatinga against melanoma and hepatocarcinoma

Lorenza Andres Ameida De Souza, Yuri de Oliveira, Diego Menezes, Alene Vanessa Santos

P29: The role of Chromogranin A as a biomarker in drug resistant neuroblastoma

Ahmad Zaki Asraf, Raymond Stallings, Olga Piskareva, Ross Conlon

P30: Membrane sweep at term gestation in CUMH; a case-control study

Siún Sweeney-Landers, Cathy Burke

P31: Study of the variability of glucose levels in patients with diabetes undergoing continuous glucose monitoring

Paraic Behan, Seamus Sreenan

P32: Inflammatory cytokine response to decreased plasma alpha-1 antitrypsin levels in individuals with the MZ genotype

Ahmed Organjee, Tatsiana Crosbie-Staunton, Emer Reeves, Noel McElvaney

P33: Analysing the role of SRC-1 in breast cancer stem cell formation and activity

Crystal Mieres, Leonie Young, Sara Charmsaz

P34: Screening Streptococcus pneumoniae isolates for virulence genes

Aya Al-Jalamdeh, Mary Corcoran, Martha McElligott, Niall Stevens, Hilary Humphreys

P35: Assessment of the relevance of admission clerking criteria taught to medical students at King Abdulaziz University to real hospital practice

Rashid Barnawi, Abdulaziz Ghurab, Sultan Alfaer, Hassan Balubaid, Kamal Hanbazazah, Mohammed Bukhari

P36: Pattern of emergency department visits during Hajj period

Mohammed Alsakkaf, Ahmad Mirza, Amrallah Mohammed

P37: Anti-Dengue activity of Aspergillus terreus (sulochrin); An in vitro study

Anastasia Pratanata, Maria Nathania, Tsabita Annisa, Beti Dewi

P38: The comorbidome in alpha-1 antitrypsin deficiency

Kuok Zhen Lee, Tomas P. Carroll, Laura Fee, Noel G. McElvaney

P39: MLO-Y4 cells behave more like osteocytes in response to mechanical stimulation when cultured in 3D

Rachel C. White, Robert T Brady, Fergal O’Brien

## O1: Assessing the protective effect of dexrazoxane against doxorubicin-induced toxicity in HL-1 cardiomyocytes

### Yvonne Sweeney, Hugh O’Neill, Garry Duffy

#### Royal College of Surgeons in Ireland, Dublin, Ireland

##### **Correspondence:** Garry Duffy – Royal College of Surgeons in Ireland, Dublin, Ireland

**Introduction:** Doxorubicin (DOX) is an anthracycline that is used for a wide range of malignant conditions. However its off-target effect causes cardiotoxicity. Dexrazoxane (DEX) is the only clinically approved cardioprotective agent against anthracycline toxicity. Its activity has been attributed to its iron-chelating effects. The aim of this project was to assess the protective effect of DEX against DOX-induced toxicity in an HL-1 cardiomyocyte model, and to investigate an early stage marker involved in cellular damage by DOX.

**Methods:** HL-1 cardiomyocytes were cultured for the purpose of bioactivity studies. The half maximal inhibitory concentration (IC-50) of DOX was established. Then the ability of DEX to recover damaged cells was assessed using measures of cell viability. A variety of DEX concentrations with HL-1 s were studied in vitro. Finally, an early stage marker involved in cellular damage by DOX was examined. An assay kit was used for the study of dsDNA breaks through the detection of γ-H2AX - a phosphorylated histone historically proven as a highly specific and sensitive molecular marker for dsDNA damage detection.

**Results:** The IC-50 of DOX was 3 μM. When DEX was combined, there was an additional toxic effect on HL-1 s. The inhibitory effect of DEX on cell viability ceased at 10 μM. The γ-H2AX assay showed decreased dsDNA breaks in cells treated with DEX compared with those treated with DOX alone. The dsDNA breaks were increased in cells treated with DOX alone compared with control (cells alone) (P < 0.05), and dsDNA breaks were increased in cells treated with DOX alone versus those treated with combined DOX and DEX (P < 0.05).

**Discussion:** DEX was found to abolish the DNA damage signal γ-H2AX caused by DOX in HL-1 s as demonstrated in the γ-H2AX assay, suggesting an alternative mechanism of cardioprotective action of DEX.

## O2: Role of KCNQ1 in epithelial barrier repair

### Daniel Creegan^1^, Viviana Bustos^2^, Brian J. Harvey^2^

#### ^1^Royal College of Surgeons in Ireland, Dublin, Ireland; ^2^Department of Molecular Medicine, Royal College of Surgeons in Ireland, Beaumont Hospital, Dublin, Ireland

##### **Correspondence:** Brian J. Harvey – Department of Molecular Medicine, Royal College of Surgeons in Ireland, Beaumont Hospital, Dublin, Ireland

**Background:** The potassium channel KCNQ1 has been identified as a tumor supressor in mouse and human colorectal cancer tissues and as a β-catenin partner at the plasma membrane [1,2]. Transcriptional Factor 4 (TCF-4) forms a complex with its coactivator β-catenin and plays an important role in carcinogenesis of colonic epithelium [2,3].

The aim of this study was to evaluate the involvement of TCF4: β-catenin KCNQ1 signaling pathway in modulating the integrity of the epithelial barrier in colorectal cancer (CRC) cell lines DLD-1 and HT29cl.19A which are representative of the intermediate and well-differentiated CRC phenotype, respectively.

**Methods:** A wound-healing assay was used to study the repairing ability of the epithelial monolayer at time points 24 and 48 h post injury, following the inhibition or activation of the TCF4: β-catenin signaling complex.

The DLD-1 cell line expressed low levels of KCNQ1 and inhibition of TCF-4 in this cell line restored some characteristics of a more differentiated CRC phenotype. DLD-1 cells were transfected with a plasmid carrying a genotype dominant negative for TCF-4 (hΔN-TCF4) to address the relationship between TCF4 inhibition, KCNQ1 expression and epithelial wound repair.

To explore the role of β-catenin/TCF4 complex during wound healing repair in HT29cl19a cells, β-catenin activity was increased through the inhibition of glycogen synthase 3-β (GSK3-β) with the GSK3-inhibitor GSK-3iX.

Western Blotting was used to detect KCNQ1 and N-Cadherin proteins in both cell lines following pharmacological treatments or plasmid transfection.

**Results:** The KCNQ1 expression and the rate of wound closure after injury was found to be enhanced in DLD-1 cells transfected with the hΔN-TCF4 plasmid which reduced the TCF4 transcriptional activity. Inhibition of β-catenin expression and function using GSK-3iX inhibited wound closure rate in in HT29cl.19A cells after injury which was associated with decreased KCNQ1 expression and increased N-cadherin expression.

**Conclusion:** These results indicate that KCNQ1 expression, regulated by the β-catenin:TCF-4 signaling complex is critical for epithelial barrier repair following a wound injury. We propose that KCNQ1 may be a promising new therapeutic target in restoring epithelial barrier function in inflammation or injury.

## O3: The suitability of non-small cell lung cancer cytology preparations for the analysis of anaplastic lymphoma kinase gene rearrangements

### Alexander Dalphy^1^, Anthony O’Grady^2^, Elaine Kay^2^

#### ^1^Royal College of Surgeons in Ireland, Dublin, Ireland; ^2^Department of Pathology, Beaumont Hospital, Royal College of Surgeons in Ireland, Dublin, Ireland

##### **Correspondence:** Elaine Kay – Department of Pathology, Beaumont Hospital, Royal College of Surgeons in Ireland, Dublin, Ireland

**Introduction:** It has been demonstrated that patients with non-small cell lung cancer (NSCLC) bearing an anaplastic lymphoma kinase (ALK) gene rearrangement benefit from targeted therapies, which offer improved treatment response rates and overall survival when compared to traditional chemotherapy. To date in Ireland, most ALK testing has been performed on formalin-fixed paraffin embedded (FFPE) tissue and cell blocks. Approximately 13 % of these blocks lack sufficient material for analysis, leaving some patients without an important ALK gene rearrangement result. The number of cases with insufficient material for analysis could be decreased substantially if testing could be performed on NSCLC cytology preparations, which are routinely taken from the same lesion while collecting the initial specimen for FFPE processing. In this retrospective study, the suitability of cytology preparations for ALK testing was examined by determining testing success rates and result concordance with corresponding FFPE samples.

**Methods:** Sixteen NSCLC adenocarcinoma cytology preparations were tested for ALK gene rearrangements by fluorescence in-situ hybridisation (FISH). These samples consisted of three different preparations: ThinPrep preparations (n = 11), direct smears (n = 4), and a cytospin preparation (n = 1). The preparations selected were evaluated beforehand to ensure adequate tumour cellularity for testing and that corresponding FFPE samples were previously FISH tested. Representative images were taken of all samples tested to maintain a permanent record and allow future review of test results.

**Results:** ALK FISH was successful in 9 of the 11 ThinPrep preparations (82 %), but was unsuccessful in all direct smear and cytospin preparations. The successful ThinPrep cases all matched the original FFPE result, demonstrating 100 % concordance.

**Discussion:** The results of this study show that ThinPrep cytology preparations are suitable for ALK FISH testing if insufficient FFPE material is available. Direct smears and cytospin preparations are not currently suitable. The ability to use ThinPrep slides for testing will help ensure all patients receive an ALK result, which can improve their prognosis. Given the limited sample sizes in the study, it is recommended that more samples of all three preparation types are evaluated.

## O4: Penetrating keratoplasty and descemet’s stripping automated endothelial keratoplasty may lead to deterioration in glaucoma management

### Katarzyna Samelska, Justyna Izdebska, Anna Kurowska

#### Department of Ophthalmology, Second Faculty of Medicine, Medical University of Warsaw, Poland

##### **Correspondence:** Anna Kurowska – Department of Ophthalmology, Second Faculty of Medicine, Medical University of Warsaw, Poland

**Introduction:** Keratoplasty is a treatment for corneal diseases such as pseudophakic bullous keratopathy (PBK) and Fuchs’ dystrophy.

Penetrating keratoplasty (PK) has been in use for over 100 years and is applicable as a treatment for disorders found in every layer of cornea. Descemet’s stripping automated endothelial keratoplasty (DSAEK) is a newer transplant technique in which only the posterior corneal tissue is replaced.

PK and DSAEK may be complicated by postoperative glaucoma in healthy eyes or lead to glaucoma deterioration in eyes previously treated for glaucoma.

The aim of the study was to determine whether PK or DSAEK leads to greater deterioration in the management of preexisting glaucoma.

**Methods:** The research is a retrospective study based on documentation of the patients diagnosed with Fuchs’ dystrophy or PBK who underwent DSAEK or PK in 2009–2013 and had been diagnosed with glaucoma prior to the keratoplasty. Patients observed for a time shorter than 12 months or qualified for retransplantation were excluded from the study.

The patients’ assessment during the 12-month observation was recorded. Glaucoma deterioration was assessed based on two factors: an increase in the number of anti-glaucoma medication types prescribed as well as any anti-glaucoma surgeries performed.

**Results:** Out of 22 eyes that underwent PK, there was an increase in the number of anti-glaucoma medication types prescribed in 4(18 %) cases during the first 12 months following keratoplasty. Aditionally, no anti-glaucoma surgeries were performed in any of the cases during these 12 months.

Out of 10 eyes that underwent DSAEK, there were anti-glaucoma surgeries performed in 2 cases and increase in medication types prescribed in 1 case during these 12 months. Overall, a deterioration in glaucoma management was noted in 3(30 %) cases.

12 months after the surgery, there was a decrease in number of anti-glaucoma medication types prescribed in 6 cases: all of them after PK.

There was a higher incidence of glaucoma deterioration in DSAEK group compared to PK group.

**Discussion:** DSAEK may present a higher risk of glaucoma deterioration than PK. It is an interesting finding, given that previous studies implicate that DSAEK (compared to PK) does not have an increased risk of glaucoma in eyes with no prior history of glaucoma. The findings above should be taken into consideration when qualifying the patients for these procedures.

## O5: The effect of Resolvin D1 on normal and cystic fibrosis human bronchial epithelium

### Rena Al-Zubaidy^1^, Magdalena Mroz^1^, Brian Harvey^2^

#### ^1^Royal College of Surgeons in Ireland, Dublin, Ireland; ^2^Department of Molecular Medicine, Beaumont Hospital, Royal College of Surgeons in Ireland, Dublin, Ireland

##### **Correspondence:** Brian Harvey – Department of Molecular Medicine, Beaumont Hospital, Royal College of Surgeons in Ireland, Dublin, Ireland

**Introduction:** Cystic fibrosis (CF) is an autosomal recessive disease which involves a malfunctioning epithelial chloride ion channel (CFTR) and a hyper-stimulated Na + channel (ENaC), this malfunction eventually leads to a reduction in the height of the airway surface liquid layer (ASL), ciliary dyskinesis, thickening of a viscous mucus layer overlying epithelial cells resulting in defective mucociliary clearance and chronic bacterial infection. Recent research has highlighted potential therapeutic effects of pro-resolution immunomodulatory molecules in the pathology of CF. This project tested the hypothesis that the new immunomodulatory molecule Resolvin D1 can restore ASL height and ion transport in CF bronchial epithelium.

**Methods:** The effects of Resolvin D1 on ASL height and ion transport were investigated in NuLi-1 (normal genotype) and CuFi-1 (CF genotype, Δ508/Δ508) primary immortalized airway epithelial cells were grown under an air-liquid interface. Confocal fluorescence microscopy was used to measure the ASL height. Electrogenic transepithelial ion transport was measured using the short-circuit current (Isc) technique in cell monolayers mounted in Ussing chambers.

**Results:** Analysis of the images from the confocal microscope showed that Resolvin D1 increased ASL height in cystic fibrosis bronchial epithelium (n = 3), but not in normal epithelium (n = 2). Na + and Cl- transport was measured in Ussing chambers experiments by addition of amiloride an ENaC channel blocker or bumetanide, Na + −K + −Cl--cotransporter inhibitor, respectively. We found out that Resolvin D1 have no effect Cl- secretion in either NuLi-1 (n = 4) nor CuFi-1 cells (n = 1). The absence of a Cl- secretory response in Nuli and CuFi cells may indicate that RvD1 increases ASL height through effects on ENaC rather than CFTR or to activate an alternative non-CFTR Cl- channel. As the statistics are too low to conclude if RvD1 affected ENaC in CuFi-1 cells, more experiments in CF bronchial epithelium are needed to test this hypothesis.

**Discussion:** The results from these experiments show that Resolvin D1 increases ASL height in CF bronchial epithelial cells. Further studies are warranted to determine the molecular mechanisms involved in restoring ASL dynamics. Resolvin D1 may have potential as a new therapy to restore mucocilairy clearance in CF airway lung disease.

## O6: Validity of clinical assessment compared with plantar fascia thickness on ultrasound for plantar fasciitis: a cross-sectional study

### Ryan Fagan^1^, Helen French^1^, Vanessa Cuddy^2^, Jennifer Ashton^2^, Michelle Clarke^2^

#### ^1^Department of Physiotherapy, Royal College of Surgeons in Ireland, Dublin, Ireland; ^2^Department of Physiotherapy, Beaumont Hospital, Dublin, Ireland

##### **Correspondence:** Michelle Clarke – Department of Physiotherapy, Beaumont Hospital, Dublin, Ireland

**Introduction:** Plantar fasciitis (PFS) is a degeneration of the plantar aponeurosis in the foot. Ultrasound (US) can be used as a diagnostic imaging technique for this condition, with similar diagnostic accuracy to Magnetic Resonance Imaging (MRI). Common factors associated with PFS include increase Body Mass Index (BMI), reduced dorsiflexion of the ankle and pronation of the midfoot. This study aimed firstly to determine the diagnostic utility of the clinical diagnosis of PFS compared with US examination, and secondly, to determine the relationship between a range of symptom-related and physical examination items and US-diagnosed PFS.

**Methods:** This cross-sectional study was approved by the Ethics (Medical Research) Committee Beaumont Hospital (REC 14/54). Patients referred from orthopaedic and rheumatology clinics were screened for eligibility and informed consent was obtained. Clinical criteria were based on presence of medial heel pain for a minimum of six weeks, aggravated by rising or initial weight bearing after inactivity. Study participants underwent clinical and US examination by two independent blinded assessors. PFS was determined on US by measuring plantar fascia thickness. The following characteristics were recorded: BMI, foot type (pronated, supinated, neutral) and ankle dorsiflexion range. Diagnostic accuracy was determined by estimating sensitivity and specificity of clinical criteria against US measurement (gold standard). Mann-Whitney U- tests were used to compare differences in symptom-based and physical examination variables between those with and without PFS.

**Results:** Fourteen participants (28 feet) were recruited. Sensitivity and specificity of clinical diagnosis was 62.50 % (95 % CI: 35.43 % to 84.80 %) and 58.33 % (95 % CI: 27.67 to 84.23 %), respectively. Increased body weight was significantly associated with PFS (r = 0.44; p = 0.04). 75 % of those with US-diagnosed PFS had altered biomechanics compared to 92 % of those who had negative US. Reduced ankle dorsiflexion was present in 87.5 % of US-diagnosed PFS patients, and 83.3 % of those with a negative diagnosis of PFS.

**Discussion:** Clinical diagnosis demonstrated only moderate diagnostic accuracy for PFS. Weight was significantly associated with PFS. Foot type and reduced dorsiflexion may have an association with foot pain, but not PFS alone: Small sample sizes may partially explain the results: Clinical assessment alone may not be sufficient in ruling in or ruling out PFS. Further clinical criteria may have to be considered to aid in identification of PFS for targeted treatment and to aid recruitment in future PFS research.

## P1: Undergraduate medical research in Gulf Cooperation Council (GCC) countries: A descriptive study of students’ views

### Zaid Sayedalamin^1^, Mukhtiar Baig^2^, Osama Almutairi^1^, Hassan Allam^1^,Taher F. Halawa^3^, Hazem M. Atta^2^

#### ^1^Rabigh Faculty of Medicine, King Abdulaziz University, Jeddah, Saudi Arabia; ^2^Department of Clinical Biochemistry, Rabigh Faculty of Medicine, King Abdulaziz University, Jeddah, Saudi Arabia; ^3^Department of Pediatrics, Rabigh Faculty of Medicine, King Abdulaziz University, Jeddah, Saudi Arabia

##### **Correspondence:** Hazem M. Atta – Department of Clinical Biochemistry, Rabigh Faculty of Medicine, King Abdulaziz University, Jeddah, Saudi Arabia

**Introduction:** There is a lack of research-oriented physicians in several Asian countries and especially in Gulf region countries. In this context, it is important to explore medical students’ perceptions and motivations towards research. The aim of the present study was to investigate research attitude, practices, and motivations among medical students from GCC countries.

**Methods:** The present cross-sectional study was carried out during the Ninth International Scientific Conference for Medical Students in the GCC Countries in Alain, United Arab Emirates, in December 2014. An anonymous, cross-sectional, self-report questionnaire was administered to medical students who attended the conference.

**Results:** There were 228 students who participated in this study, among them 88(38.6 %) were males. Most of the students 38 % were participating from Saudi Arabia, 20.6 % from UAE, 17.1 % from Oman, 12.7 % from Kuwait and 11.4 % from Bahrain. Among participants, 43.0 % had experience of funded research and 53.1 % had a contribution in research, which either had a social impact or provided an industrial application. The confidence of participants about their ability to interpret and to write a research paper was quite high (70.2 %). The majority of the students (87.3 %) believed that undergraduate student can conduct research and can present in conferences, but still there were only 31.1 % who believed that they can do it without supervision. Improving research skills, attaining research publications, and improvement in patient care were claimed the top three motives behind conducting research. It was important to note that, the majority (75.0 %) were compelled to research for facilitating their acceptance to a residency program and 63.6 % due to compulsion for research methodology course.

**Discussion**: The half of the students reported strong involvement in research activities. The reported knowledge and attitudes among the medical students considered high.

## P2: Positive fluid balance as a prognostic factor in children with sepsis during first 3 hours of resuscitation in intensive care unit

### Linda Sirone, Renars Erts, Jana Pavare

#### Riga Stradins University, Riga, Latvia

##### **Correspondence:** Jana Pavare – Riga Stradins University, Riga, Latvia

**Introduction:** Sepsis is a severe and life threatening condition where fluid infusion can be lifesaving, especially for initial treatment. Only a few studies had been done performed about fluid infusion management in children, yet it’s an everyday clinical challenge. Several studies have correlated positive fluid balance with higher mortality in adults. The aim of our study was to evaluate the correlation between positive fluid balance during the first three hours of resuscitation with reduced survival in children with sepsis.

**Methods:** In retrospective study medical records of 48 children admitted to PICU of Children’s Clinical University Hospital of Latvia with sepsis during the period of 2009–2015 were examined. Fluid intake and output were measured and the balance was calculated for first 3 hours of resuscitation, it was then compared between survivors and nonsurvivors. To evaluate the results ROC curve analysis and unpaired t-test was performed. P-value <0.05 was used as statistical significance.

**Results:** In comparison mortality rate was higher in patients who had positive fluid balance during the first three hours of resuscitation (AUC = 0.72; 95 % CI: 0.55-0.89; p = 0.03). Median age of survivors and nonsurvivors and the time spent for their stay in intensive care unit did not statistically differ (p > 0.05).

**Discussion:** Findings suggest that a positive fluid balance during the beginning of resuscitation in children with sepsis may be a trustworthy prognostic factor of higher mortality, further research and larger studies are needed to be performed for stronger approval.

## P3: Patients on aspirin: Too little or too much?

### Louis Richter^1,2^, Joseph Morris^1,2^, Irene Oglesby^2,3^, Eimear Dunne^2,3^, Dermot Kenny^2,3^

#### ^1^Royal College of Surgeons in Ireland, Dublin, Ireland; ^2^Molecular and Cellular Therapeutics, Royal College of Surgeons in Ireland, Dublin, Ireland; ^3^Clinical Research Centre, Royal College of Surgeons in Ireland, Dublin, Ireland

##### **Correspondence:** Dermot Kenny – Molecular and Cellular Therapeutics, Royal College of Surgeons in Ireland, Dublin, Ireland

**Introduction:** It is not known if aspirin is effective in patients with coronary artery disease. Light transmission aggregation (LTA) is used as a measure of aspirin effect but it has limitations. To determine the physiological response of platelets in patients taking aspirin, we assessed platelet function using the novel Dynamic Platelet Function Assay (DPFA) against the current gold standard test, LTA.

**Methods:** Blood from 307 patients taking aspirin only was perfused under arterial shear (1,500 s-1) through a custom-built flow chamber coated with human von Willebrand Factor (VWF). Platelet activity was recorded by digital-image microscopy and then processed.

**Results:** All of the patients had an “appropriate” aspirin response as defined by LTA, that is aggregation < 20 %. In contrast there was a marked heterogeneity of results using the DPFA. The interquartile ranges (IQR) are as follows: Platelet tracks (number of platelets that move a distance greater than 1.5x their radius) IQR: 259.0–509.0, stably adhered platelets (number of platelets that have moved less than 1.5 times their average radius from their start to end position as a singlet only) IQR: 128.0–242.0, speed of platelets (weighted median velocity of translocating platelets, μM/sec) IQR: 4.32388–8.52876, arrested translocators (number of platelets that have adhered in a stationary manner for at least 100 frames to the VWF-coated surface) IQR: 52.0–83.0.

**Discussion:** The DPFA is able to provide parameters of measurement in the physiological response of platelets. The LTA is only able to measure the biochemical response which is not an accurate reflection of platelet behaviour in vivo. Our results demonstrate for the first time a marked physiological heterogeneity in platelet response. This suggests that some patients may benefit from additional aspirin whereas others have an excessive dose.

## P4: Beta catenin/TCF4 activation reduces KCNQ1 current in colonic monolayers

### Ibrahim Mohammed Mahdi Khayyat^1^, Viviana Bustos Salgado^2^, Brian J. Harvey^2^

#### ^1^Royal College of Surgeons in Ireland, Ireland, Dublin; ^2^Department of Molecular Medicine, Beaumont Hospital, Royal College of Surgeons, Dublin, Ireland

##### **Correspondence:** Brian J. Harvey – Department of Molecular Medicine, Beaumont Hospital, Royal College of Surgeons, Dublin, Ireland

**Background:** Colorectal cancer (CRC) is the third most common cancer with the fourth highest mortality rate worldwide. There is evidence that a relationship exists between the expression and activity of a potassium (K^+^) ion channel, encoded by *KCNQ1* gene, and pre-tumour cell hyperplasia and colorectal cancer development. β-catenin is a widely expressed protein involved in the transcriptional regulation of many genes. Once translocated to the nucleus, β-catenin acts as a co-factor for T-cell Factor 4 (TCF-4) leading to the activation or repression of gene transcription. The aberrant activation of this pathway has also been reported in colorectal cancer. A link between nuclear β-catenin and KCNQ1 function has not previously been reported in colorectal cancer. The goal of this study was to explore if β-catenin nuclear translocation modifies KCNQ1 potassium channel epithelial function in colorectal cancer cells.

**Methods:** Confluent monolayers of cl.19A cells were grown on semi-permeable supports until transepithelial resistance (TER) reached 1 kg*cm^−2^. The inserts were mounted in Ussing chambers and basolateral membrane K^+^ conductance was measured by permeabilizing the apical membrane. An apical-to-basolateral K^+^ gradient was imposed. Ouabain was added basolaterally to inhibit Na^+^-K^+^-ATPase pump activity. In these conditions, changes in I_*sc*_ are wholly reflective of changes in basolateral K^+^ conductance (I_K_). Significant differences were calculated by ANOVAwith a *P* value of <0.05 regarded as statistically significant. Nuclear β-catenin levels were enhanced by treatment with GSK-3 iX for 24 h, which activates β-catenin/TCF4 complex. Western blot analysis was used to confirm nuclear localisation of β-catenin.

**Results:** Forskolin-stimulated KCNQ1 currents were measured in untreated cl.19A monolayers. Treatment with GSK-3 iX decreased the forskolin-stimulated KCNQ1 currents. Activation of β-catenin/TCF4 complex showed no significant differences on the calcium activated intermediate conductance IK1 (KCNN4) currents activated by muscarinic agonist, carbachol. Thus the decreased current observed in response to GSK-3 iX treatment is specific to KCNQ1.

**Conclusion:** Translocation of activated β-catenin into the nucleus inhibits KCNQ1 epithelial function. This is the first functional study to associate β-catenin localisation with KCNQ1 activity in a disease state and enhances our understanding of colorectal cancer.

## P5: Size Matters. Abdominal aortic aneurysm: adherence to surveillance imaging guidelines

### Jonavan Tan^1^, Daragh Moneley^2^, Austin Leahy^2^, Patricia Fitzgerald^3^

#### ^1^Royal College of Surgeons in Ireland, Dublin, Ireland; ^2^Department of Surgery, Beaumont Hospital, Royal College of Surgeons in Ireland, Dublin, Ireland; ^3^Non Invasive Vascular Unit, Beaumont Hospital, Royal College of Surgeons in Ireland, Dublin, Ireland

##### **Correspondence:** Patricia Fitzgerald – Non Invasive Vascular Unit, Beaumont Hospital, Royal College of Surgeons in Ireland, Dublin, Ireland

**Introduction:** Abdominal aortic aneurysm (AAA) is a common, potentially life-threatening condition. The major risk of AAA is rupture, which increases with increasing aneurysm diameter. Regular surveillance (usually abdominal ultrasound) at suitable intervals guides appropriate timing of surgical or endovascular intervention. Apparent discrepancies in imaging follow-up raised concerns regarding patient safety and clinic efficiency. A dedicated Aortic Surveillance Clinic (ASC) was proposed. AAA patients from one of the 3 Vascular Surgical OPD clinics (VSC) were referred to the ASC, here patients would be reviewed by a Vascular Physician directly following ultrasound examination. Planned return to Vascular Surgeon occurred when the AAA reached appropriate size for intervention consideration. The aim of this study is to analyse adherence rates of aortic imaging requests against guidelines, to determine if a dedicated clinic achieves greater adherence than the current Vascular Surgical OPD model.

**Methods:** Patient appointment lists (12 clinics, March-June 2015) from two VSCs (1&2) and the ASC were retrospectively reviewed to identify AAA patients. Scheduling of aortic imaging intervals was analysed to determine adherence to surveillance guidelines for previous visits, allowing a one-month tolerance.

**Results**: 1102 patients attended these clinics during the study period, with a diagnosis of AAA in 165 VSC and 46 ASC patients. Of these, 79 and all 46 patients, respectively, had no previous AAA intervention, and so were eligible for surveillance. These 125 patients had 621 episodes in total. Only 26 % and 29 % of patients attending VSC1 and VSC2, respectively, had imaging scheduled per guidelines, compared to almost 90 % in the ASC. Patients with larger (high-risk) aneurysms had poorer attendance rates overall, with no VSC1, and only 27 % of VSC2 patients adhering to guidelines, versus 80 % in the ASC. Non-attendance at scheduled appointments was not a significant contributor (2.7 %).

**Discussion:** There is no apparent uniform agreement on surveillance guidelines at VSCs, with variable timing of imaging requests. Possible explanations include: lack of clearly defined parameters, failure to clearly communicate or disseminate information, and/or failure to fully appreciate clinical relevance of appropriate surveillance.

**Conclusion:** A dedicated clinic appears more efficient, with significantly higher scheduling adherence rates. Implication: Clarification of AAA surveillance intervals with dissemination of information at team changeover times is required, with planned future re-audit. Continuation/expansion of the ASC appears indicated, but requires cost-benefit analysis. Retrospective review of surveillance prior to AAA rupture would likely clarify patient safety impact.

## P6: Endoscopic retrograde cholangiopancreatography in the west of Ireland: Procedural outcomes and peri-procedural complications

### Evelyn Fennelly^1^, Grace Harkin^2^, John Lee^2^

#### ^1^School of Medicine, National University of Ireland, Galway, Ireland; ^2^Department of Gastroenterology, University Hospital Galway, Galway, Ireland

##### **Correspondence:** John Lee – Department of Gastroenterology, University Hospital Galway, Galway, Ireland

**Introduction:** The aim of our study was to evaluate ERCP procedural outcomes and peri-procedural complications in a tertiary centre in the West of Ireland.

**Methods:** Patients who underwent ERCP at our institution from January 2012 to June 2015 were included. Retrospective demographic and procedural data was collected using a standardised data collection form from a combination of endoscopy and radiology reports, discharge summaries and laboratory results. 62 patient charts were evaluated for more information. Statistical analysis was performed using SPSSv21.

**Results:** 430 ERCP’s were performed on 348 patients during the study period. The mean age was 64.03 years and 50.0 % were female.

The most common indication was choledocholithiasis (30.0 %). Duct cannulation was obtained in 94.87 % of procedures. 74.59 % of procedures involved interventions, including sphincterotomies (44.88 %) and biliary stent insertion (20.9 %). 74.47 % procedures were considered successful in dealing with the presenting clinical indication.

The overall complication rate was 12.3 %: PEP 7.9 %, oxygen desaturation 1.6 %, self-limiting bleeds 1.6 %, minor bleeds 0.7 %, perforations 0.5.%, sepsis 0.2 %, acute kidney injury 0.2 %. There were no haemorrhages, cardiopulmonary events, contrast allergy reactions, or intra-procedural deaths. There was one peri-procedural death unrelated to the procedure.

Patients who experienced PEP were younger than the cohort average (58.56 years) and mostly female (62.9 %) but neither was statistically significant. The most common indication in PEP patients was choledocholithiasis (37.1 %) but was not significant. The mean post-ERCP peak amylase value was markedly higher for the PEP patient group (1434.23 ± 987.92u/L) than the cohort average (369.6 ± 712.4u/L). Success of the procedure was significant (p 0.0009). A partially successful procedure was associated with a 5.7-fold increased risk of PEP (p 0.002), compared to a successful ERCP. An unsuccessful procedure increased the risk of PEP by 1.77-fold (p 0.377) compared to successful ERCP.

**Discussion:** Procedural outcomes for ERCP were acceptable. Overall complication rate was 12.3 %. PEP was the most common peri-procedural complication, consistent with larger multicentre studies. An unsuccessful procedure increased the risk of PEP by 1.77-fold (p 0.377) whereas a partially successful procedure was associated with a 5.7-fold increased risk of PEP (p 0.002), compared with a successful ERCP. We postulate the lower risk with unsuccessful ERCP’s may be due to less manipulation and early termination of the procedure and the increased risk with partially successful ERCP’s reflects difficult and complicated cases.

## P7: The effect of the extracellular redox environment on polyamine-platelet interactions

### Erica O’Sullivan^1^, Brian Kirby^2^, Sarah O’Neill^3^

#### ^1^Department of Medicine, Royal College of Surgeons in Ireland, Dublin, Ireland; ^2^School of Pharmacy, Royal College of Surgeons in Ireland, Dublin, Ireland; ^3^MCT Department, Royal College of Surgeons in Ireland, Dublin, Ireland

##### **Correspondence:** Sarah O’Neill – MCT Department, Royal College of Surgeons in Ireland, Dublin, Ireland

**Introduction:** Platelets play an important role in homeostasis and are implicated in CVD disease, particularly in response to changes in the extracellular redox environment. Polyamines, especially Spermine, which is important in cell growth and proliferation, have the ability to inhibit platelet aggregation. We examined how this effect changed in response to a change in the extracellular redox environment. To determine if spermine inhibits platelets in a reduced, mean and oxidised redox environment.

**Methods:** Platelets were isolated from whole blood from healthy volunteers. Ethical committee approval was gained from RCSI. Redox potentials were created using serial dilutions of GSH/GSSG and Cys/CySS. Platelet aggregometry was carried out to test thrombin-induced platelet aggregation in the presence of 1 mM Spermine and how this changes in Oxidised (−10 mV;+4 mV), Mean (−130 mV;-82 mV) and Reduced (−264 mV; −148 mV) redox environments. Results were recorded at for at least 5 minutes after the addition of thrombin (0.1U/ml).

**Results:** Aggregation reduced in the presence of Spermine (n = 13; P = 0.0057). However this % Aggregation with Spermine and thrombin increased in the presence of reduced (−264 mV) GSH/GSSG (n = 4; P < 0.5) and (−148 mV) Cys/CySS (n = 3; P > 0.5). No significant changes were noted with the oxidized (−10 mV;+4 mV) and mean (−130 mV; −82 mV) redox couples for Glutathione (n = 4; p > 0.5) and Cysteine (n = 3; p > 0.5) respectively.

**Discussion:** Changes in the extracellular redox environment affect Polyamine-platelet interactions. Spermine’s ability to inhibit platelets was reduced/overcome following a change in the Redox environment. Spermine no longer inhibited platelets in a reduced Redox environment. As the levels of polyamines may rise in cancer and the redox environment in the body may change; the effect of a redox environment on polyamines and platelets is an important consideration as this may affect homeostasis.

## P8: Functionalized gold nanoparticles: preliminary data on in vitro toxicity and comparative photothermal effect

### Diana Gonciar^1^, Teodora Mocan^2^, Cristian Matea^2^, Lucian Mocan^2^, Cornel Iancu^2^

#### ^1^Department of Medicine, University of Medicine and Pharmacy Iuliu Hatieganu, Cluj-Napoca, Romania; ^2^Regional Institute of Gastroenterology and Hepatology, Cluj-Napoca, Romania

##### **Correspondence:** Cornel Iancu – Regional Institute of Gastroenterology and Hepatology, Cluj-Napoca, Romania

**Introduction:** Nanotechnology represents an immense potential for human medical applications. Amongst the different experiments that have been imagined, including therapy and diagnosis procedures for a wide variety of diseases, the antitumor applications represent the focus of many research teams worldwide. Photothermal effect, induced by the property of nano-sized structure to transform light energy into thermal energy when exposed to laser irradiation represents a special feature enabling thermal destruction of tumor cells. However, directing the treatment towards cancer cells while leaving the healthy cells unaffected represents the major key element in generating a safe and effective therapy. As a response to this problem, binding of selectivity molecules onto the surface of nanoparticles has been proposed for enabling their selective agglomeration on the tumor cells. Nevertheless, data on comparative toxicity of the different solutions is still scarce, and there are limited comparative photothermal effect efficiency studies with newly designed nanostructures.

**Methods:** We aimed to compare toxicity and antitumor efficiency of three types of nanostructures: 1) pristine gold nanoparticles; 2) gold nanoparticles functionalized with epidermal growth factor; 3) gold nanoparticles functionalized with arginine. HepG2 cell line (hepatocellular carcinoma) was used for in vitro testing. Viability/proliferation was assessed by means of MTT assay. AnnexinV -cy3 staining was used for apoptosis evaluation, followed by fluorescence microscopy imaging procedures. For photothermal effect, laser irradiation was performed using a 808 nm 2 W laser. Pre and post-evaluation of viability and apoptosis were measured. For each of the nanostructures, 50microg/mL and 25microg/mL concentrations were used for exposure, as well as cell culture media alone (control group).

**Results:** MTT assay revealed a dose-dependent decrease in viability following 6 hours of exposure. Exposed groups had lower levels of viability/proliferation as compared to control group. However, none of the experimental groups decreased below 85 % viability and differences between any two groups remained insignificant.

Laser irradiation induces significant decrease in viability as compared to non-irradiated groups. The effect was stronger for higher concentration of each nanomaterial. Amongst the three nanostructures, the highest efficiency (p < 0.05) was obtained for gold nanoparticles functionalized with arginine, followed by gold nanoparticles functionalized with epidermal growth factor. The least effective nanophotothermalysis was obtained for pristine-exposed groups. Measured effects were reflected in higher annexin V agglomeration, as revealed by fluorescence microscopy analyses.

**Discussion:** The three studied nanostructures present good potential for human applications in hepatocellular carcinoma (good biocompatibility). Gold nanoparticles functionalized with arginine represent the most effective nanostructure for photothermal effect-based treatment of liver cancer. Further studies need to be conducted for complete evaluation of cell-nanomaterial interaction.

## P9: Imaging proteasomal inhibition after seizures in the brain: A study into cellular activity in the hippocampus of epileptic transgenic mice

### Chloe Doran^1^, Sarah Hoolahan^1^, Tobias Engel^2^

#### ^1^Royal College of Surgeons in Ireland, Dublin, Ireland; ^2^Physiology & Medical Physics, Royal College of Surgeons in Ireland, Dublin, Ireland

##### **Correspondence:** Tobias Engel – Physiology & Medical Physics, Royal College of Surgeons in Ireland, Dublin, Ireland

**Introduction:** Epilepsy is the most common neurological condition affecting people of all ages, with an estimated prevalence of 1 % in the Irish population. It is defined as a tendency towards recurrent unprovoked seizures, which are the result of excessive neuronal activity in the brain. Critically, to date, anti-epileptic drugs are merely symptomatic without disease-modifying properties and no anti-epileptogenic treatments have been approved for use in patients. Following a seizure, cell damage occurs which might lead to the accumulation of abnormal proteins. The ubiquitin-proteasome system (UPS) functions as the cell’s major non-lysosomal mechanism for degrading unwanted proteins in damaged cells. To date, it is not known whether seizures interfere with the correct functioning of the UPS and thereby alter intracellular protein levels, and therefore also influence the process of epileptogenesis.

The following study was designed to determine the capability of a transgenic mouse model to investigate the impact of seizures on the correct functioning of the UPS.

**Methods:** Transgenic mice overexpressing ubiquitin covalently tagged to the fluorescent protein GFP (UbG767-GFP) received an intra-amygdala injection of kainic acid which induced status epilepticus (SE). These seizures were terminated 40 minutes later using a bolus dose of IV lorazepam. The mice were sacrificed at various time-points following termination of SE. Brain sections were obtained and imaged.

These samples were analysed using techniques including Western Blot, Fluoro-Jade B and DAB Peroxidase staining.

**Results:** Western blotting with c-fos showed hippocampal recruitment during status epilepticus. This was greatest in the 8 hours following SE.

Fluoro-Jade B staining showed predominant ipsilateral CA1 and CA3 hippocampal damage, with sparing of the dentate gyrus and contralateral hippocampus following seizure activity.

UbG767-GFP mice showed similar seizure susceptibility as wild-type mice.

**Discussion:** UbG767-GFP mice represent a valid model to investigate UPS dysfunctions after seizures in vivo.

## P10: Investigating the ability of the Olfactory epithelial stem cells to differentiate into glial cells by assessing cell morphology and marker expression

### Maha Alkhattab^1^, Tijna Alekseeva^2^, William Lackington^2^, Fergal O’Brien^2^

#### ^1^Department of Medicine, Royal College of Surgeons in Ireland, Dublin, Ireland; ^2^Department of Anatomy, Royal College of Surgeons in Ireland, Dublin, Ireland

##### **Correspondence:** Fergal O’Brien – Department of Anatomy, Royal College of Surgeons in Ireland, Dublin, Ireland

**Introduction:** Peripheral nerve injuries (PNI) affect millions of people worldwide. The current clinical gold standard for PNI is autologous nerve grafting. However the technique has numerous limitations including donor site morbidity, neuroma formation and lack of tissue availability. Tissue engineering has the potential to overcome these limitations as an alternative treatment for peripheral nerve injury.

**Methods:** It is known that Schwann cells play a key role in peripheral nerve regeneration. However, harvesting of the autologous Schwann cells is invasive and requires long-term culture in the laboratory prior to implantation. One potential approach involves the delivery of stem cells designed to differentiate into Schwann cells via nerve guidance conduits. In this study the focus was to investigate whether novel stem cells, olfactory neural epithelial stem cells (ONESC) have the potential to differentiate into Schwann cells. Additionally, we investigated the effect of the extracellular matrix proteins, which can be included into the conduit design, on the differentiation process. Specifically, we assessed the ability of basal membrane proteins laminin and fibronectin to enhance cell differentiation. This was assessed using immunocytochemistry and fluorescence activated cell sorting (FACS) analysis.

**Results:** Results demonstrate that ONESC were capable of expressing Schwann cell specific markers after 3 days of being induced to differentiate and up to 14 days in culture. We have also showed that both laminin and fibronectin have significant effect on differentiated Schwann cells adhesion. Fibronectin showed significantly higher number of adherent cells after 14 days in culture.

**Discussion:** Collectively, these results suggest that ONESCs is a viable source of autologous Schwann cells and fibronectin should be included in the nerve conduit design for future PNI repair applications. This research is kindly sponsored by the health research board.

## P11: Beaumont Hospital cystic fibrosis service audit and annual report

### Nabeehah Moollan^1^, Chloe Doran^1^, Noel Gerry McElvaney^1,2^, Cedric Gunaratnam^1,2^

#### ^1^Department of Medicine, Royal College of Surgeons in Ireland, Dublin, Ireland; ^2^Beaumont Hospital, Beaumont, Dublin, Ireland

##### **Correspondence:** Cedric Gunaratnam – Department of Medicine, Royal College of Surgeons in Ireland, Dublin, Ireland

**Introduction:** Cystic Fibrosis (CF) is an autosomal recessive disorder characterised by multi-organ impairment which is most prevalent in the Irish population. Beaumont Hospital (BH) is one of the main CF centres for adults in Ireland. It is therefore necessary to analyse patient data, funding required and predict patient prognosis on a yearly basis.

**Methods:** The data obtained from the BH CF database was analysed and compiled into graphs using Microsoft Excel®. It was also used to estimate annual funding requirements based on the UK CF Banding System. Patients were allocated into bands 1–5 based on the severity of their disease, a proposed tariff is set for each band, and the overall funding required was calculated based on the number of patients in each band. The CF-ABLE Score was also calculated for each patient using the CF-ABLE Score template, in order to predict patient prognosis and to analyse fluctuations in the patient’s score over time. This was repeated for 2013. A paired t-test score was calculated using the Prism Statistical Software®.

**Results:** The results obtained were similar to those in the 2013 audit, with an overall increase in patient FEV1% and a decrease in the number of IVAB required, resulting in a decrease in those in the more severe bands and those with more severe CF-ABLE scores. The paired t-test conducted showed a strong positive correlation of 0.85 and a p-value of 0.42.

**Discussion:** The total annual cost to care was lower in 2014 than 2013. If applied to the Irish healthcare system, banding could be a way of determining the funding required for the BH CF Unit to maintain their patient’s health at a high standard. In addition it highlights the fluctuations in resources required from one year to the next.

Most patients had low CF-ABLE scores predicting a good prognosis. The correlation coefficient (0.85) showed a strong positive association between the individual patient scores from one year to the next. The p-value (0.42) showed that any difference in mean patient scores from 2013 to 2014 was not statistically significant. The introduction of the CF-ABLE score to the annual audit can be used to predict patient prognosis and appropriate referral for transplant. However as the CF-ABLE score predicts patient severity over a four year period, it is recommended that a paired t-test be repeated in future audits in order to further strengthen the results found.

## P12: Quick cognitive screening: the 6-item cognitive impairment test and the temporal orientation score

### Lorraine Scanlon^1^, Noeleen Brady^2^, Suzanne Timmons^2^

#### ^1^School of Medicine, University College Cork, Cork City, Ireland; ^2^Centre for Gerontology and Rehabilitation, University College Cork, St Finbarr’s Hospital, Cork City, Ireland

##### **Correspondence:** Suzanne Timmons – Centre for Gerontology and Rehabilitation, University College Cork, St Finbarr’s Hospital, Cork City, Ireland

**Introduction:** The standardised Mini Mental State Examination (sMMSE) is a gold standard cognitive assessment, but takes 10–15 minutes to complete. The 6-CIT and TOS have been suggested as short, accurate cognitive screens, allowing routine screening of all older in-patients and hence earlier diagnosis and treatment of dementia/cognitive impairment. This study hypotheses that the 6-CIT and the TOS compare favourably to the sMMSE. The main aim of this study is to assess the accuracy of the 6-CIT and TOS compared to the sMMSE in screening for dementia in older in-patients.

**Methods:** Patient (N = 216) were selected from the Cork-IDEAS study and their records were analysed. Demographics, sMMSE, 6-CIT and TOS results were manually extracted. A subset of 22 patients underwent timed cognitive testing using the 6-CIT and TOS. All statistical analysis was carried out using IBM SPSS version 20 and MedCalc version 15.8.

**Results:** There was significant correlation between sMMSE and both 6-CIT and TOS scores (r = − 0.695, p < 0.001 and r = − 0.531, p < 0.001 respectively), strongest for the 6-CIT. The mean time taken to complete the 6-CIT was 3.03 minutes. Using previously reported cut-offs for dementia (8/9, i.e. 8 = normal; 9 = dementia), the 6-CIT had sensitivity of 79.2 and specificity of 85.6 (AUC = 0.82) compared to the accepted sMMSE score cut-off for dementia (≥24/30 = normal). As a preliminary quick screen, a cut-off of 5/6 (sensitivity 92.2, specificity 64.7) would safely avoid the need for further testing in 44 % of patients (i.e. 96/216 patients scored ≤5).

**Discussion:** Both the 6-CIT and TOS compared favourably to the sMMSE. The 6-CIT out-performed the TOS. Using “sensitive” cut-offs, the 6-CIT could prove a useful quick screening test for dementia, reducing the numbers of patients requiring a longer cognitive test in a busy hospital setting.

## P13: Granular analysis of causes of peritoneal dialysis technique failure in the first six months of therapy

### Richard Bresler^1^, Zita Abreu^2^, Stefan Trohonel^3^, Joanne Bargman^3^

#### ^1^Department of Medicine, Royal College of Surgeons in Ireland, Dublin, Ireland; ^2^Department of Nephrology, University Hospital Network, Toronto, Canada; ^3^Home Peritoneal Dialysis Unit, University Hospital Network, Toronto, Ontario, Canada

##### **Correspondence:** Joanne Bargman – Home Peritoneal Dialysis Unit, University Hospital Network, Toronto, Ontario, Canada

**Introduction:** Mechanisms of PD failure have been centered on patient characteristics including technique failure, infectious complications and patient phenotype. Early referral to a nephrologist has been shown to reduce mortality, hospitalization and increase PD uptake. An experienced PD team, structured training program, patient support and continuous quality improvement (CQI) are key factors. Nonetheless, a significant number of patients leave PD in the first six months. Most data comes from large registries, where it is often difficult to pinpoint the cause of technique failure.

This single-center retrospective study evaluated mechanisms of PD failure and PD referral patterns.

**Methods:** Retrospective clinical chart review of 58 peritoneal dialysis patients was performed at a single academic PD program. All PD patients who experienced technique failure within 6-months were included. Clinical charts were evaluated for baseline demographics, catheter insertion type, PD days, home support, training, referral origin and technique failure.

**Results:** A total of 58 patients were reviewed. Mean age of 67.1 (SD 72.3), Male sex, 39 (67.0 %), Laparascopic PD Insertion 38 (65.5 %). Referral Pattern analysis showed: Pre-Dialysis Clinic 26 (44.8), IHD 12 (20.7), HHD 1 (1.7), Other Clinics 12 (20.7), General Nephrology Clinic 7 (12.0).

Reasons for premature (<6 months) termination of PD included: Patient preference (16 %), Peritonitis (7 %), Resource Constraint (5.4 %), In-Patient Death (53 %), Site Complications (7 %), Intrathoracic Complications (9 %) and Renal Recovery (2 %). Home support analysis showed 36.2 % of patients were independent, 27.6 % had some form of nursing support and 17.2 % were ineligible for nursing support. Total PD training days, 4.777 (SD 2.13). Mean days on dialysis were 75.68 (SD 55.839) across all patients.

**Discussion:** This study demonstrates a heterogeneous population of PD patients with technique failure. Site complications, peritonitis, intra-thoracic complications and patient preference accounted for the majority of technique failure aside from patient death. Less than half of patients were seen in a pre-dialysis clinic prior to PD initiation with a large subset of patients lacking community nursing supports and PD training days. Further research into enhanced clinical care models for PD is required to address premature technique failure.

## P14: Job satisfaction of surgeons working in hajj pilgrimage: a multicenter study

### Ahmad A. Mirza^1^, Ahmed Badrek-Amoudi^2^, Rakan H. Aun^3^, Hussam A. Senan^4^, Abdulrahim A. Mirza^5^, Mohammed S. Binsaad^5^, Mian U. Farooq^6^

#### ^1^College of Medicine, Taif University, Taif, Saudi Arabia; ^2^Department of Surgery, Faculty of Medicine, Umm Al-Qura University, Makkah, Saudi Arabia; ^3^Dr. Soliman Fakeeh Hospital, Jeddah, Saudi Arabia; ^4^Department of Otolaryngology-Head and Neck Surgery, Alnoor Specialist Hospital, Makkah, Saudi Arabia; ^5^Faculty of Medicine, Umm Al-Qura University, Makkah, Saudi Arabia; ^6^Department of Strategic Planning and Institutional Advancement, King Abdullah Medical City, Makkah, Saudi Arabia

##### **Correspondence:** Mian U. Farooq – Department of Strategic Planning and Institutional Advancement, King Abdullah Medical City, Makkah, Saudi Arabia

**Background:** Hajj is one of the biggest mass gatherings in the world that requires huge organizational and material efforts including health services to the visitors. Like in every other healthcare system, the quality of care in hajj depends on several parameters, among which the well-being of clinicians and their job satisfaction. We aimed to assess the job satisfaction of surgeons working during hajj.

**Methods:** A cross-sectional multicenter study was carried out in the three major hospitals of the holy city of Makkah, Saudi Arabia. A structured questionnaire was distributed to all surgeons who were employed during hajj and their job satisfaction was compared between in-Hajj and non-hajj periods using a modified version of the Warr-Cook-Wall job satisfaction scale, which included 10 relevant items; each assessed using a 5-point likert scale starting from extremely dissatisfied (score = 1) to extremely satisfied (score = 5). Impact of their characteristics as well as other elements of job satisfaction in the questionnaire was determined through a stepwise linear regression analysis for both periods separately.

**Results:** After the questionnaire was distributed, we noticed that King Faisal hospital harbored the majority of the responded surgeons (35.7 %) and most (58.6 %) of them were aged below 40. Overall job satisfaction scores were higher in hajj period as compared to that of non-hajj, (mean ± SD = 3.72 ± 0.92 versus 3.63 ± 0.83, respectively), but the results were not statistically significant. Item-by-item analysis showed that doctors were significantly more satisfied during hajj period than non-hajj period in term of both “amount of variety in job” (*P* < 0.001) and “attention paid to suggestions” (*P* < 0.001); while they were likely to be less satisfied with regard to “hours of work” during hajj. The most significant predictor of overall satisfaction in hajj period was ‘attention paid to suggestions’, followed by ‘Physical working conditions’, ‘opportunity to use abilities’, ‘hours of work’, ‘immediate boss’ and ‘job designation’, within a six-step model explaining 91 % of the variance of the overall satisfaction score (*P* < 0.001).

**Conclusion:** The variety of works in the surgical fields and relative decision-making autonomy that characterize hajj medical service contribute significantly to promote surgeons’ job satisfaction. However, a particular attention should be paid to the physician’s adaptability to the expanded working hours, in order to avoid dissatisfaction and burnout, which may lead to suboptimal healthcare delivery to hajj pilgrims or poor clinical outcomes.

## P15: Investigation of the role of Bok using wild-type, bax-, bok-, and bax/bok-double-deficient mice

### Saheli Nandi^1^, Beatrice D’Orsi^2^, Jochen Prehn^2^

#### ^1^Department of Medicine, Royal College of Surgeons in Ireland, Dublin, Ireland; ^2^The Centre for the Study of Neurological Disorders, Royal College of Surgeons in Ireland, Dublin, Ireland

##### **Correspondence:** Jochen Prehn – The Centre for the Study of Neurological Disorders, Royal College of Surgeons in Ireland, Dublin, Ireland

**Introduction**

Excitotoxicity, over-activation of glutamate receptors, has been implicated to contribute to neuronal injury after several neurological disorders, such as stroke, trauma and seizure. The Bcl-2 family of proteins, comprising of both pro-apoptotic and anti-apoptotic members, is an essential mediator of the mitochondrial pathway of apoptosis by controlling mitochondrial outer membrane (MOM) integrity. Bok, which shares ~ 70-80 % sequence homology to Bax and Bak, has been considered part of the pro-apoptopic Bax-like subfamily. Bok is highly enriched in the CA3 hippocampal neurons and expressed in the cerebral cortex. However, no studies yet have been performed in neurons using gene deficient mice. In this summer project, we derived primary cortical neuron cultures from wild-type (WT), bax-, bok-, and bax/bok-double-deficient mice.

**Methods**

The methods used in this summer project were western blotting, to identify and quantify specific proteins and cell death assay to determine neuronal injury using propridium iodide (PI) and Hoechst 33258 staining of nuclear chromatin.

**Results**

Using western blotting analysis, we first investigated the expression of the Bax-like subfamily of proteins, including Bok, Bax and Bak, in several organs from C57BL/6 WT mice. Bok protein levels resulted abundant in the brain, kidney and spleen tissues, whereas Bax and Bak expression is constant in various tissues with Bax higher in lung and spleen, and Bak extremely elevated in lung. We next explored the expression of Bok protein in cortical neurons and astrocytes, where it was highly expressed, and microglia and BV2-like glial cells where it accumulates only marginally. In addition, we verified whether bok deficiency may affect Bcl-2 family protein expression, including Bax, and anti-apoptotic proteins, such as Bcl-2, Bcl-w, Bcl-xL and Mcl-1 in bok- and bax/bok- double-deficient cortical neurons. Lastly, to address whether bok deficiency, like bax, also protected against neuronal injury, cortical neurons from WT, bok−/− and bax−/− mice were exposed to several stimuli-induced cell death, including Staurosporine (STS), Epoxomicin, Thapsigargin. bok-deficiency failed to protect cortical neurons against STS, however showed protection against Thapsigargin-induced cell death compared to WT controls.

**Discussion**

In conclusion, findings from this summer project may help a larger study of a longer duration in understanding the role of Bok in neuronal death in greater detail. Thus, implications of this study may impact the pathogenesis of several neurological disorders, most importantly strokes, traumatic brain injuries and seizures, in which excitotoxicity is thought to have a great role.

## P16: Is it possible to predict resistance of an organism to stress based on the level of corticosterone?

### Mariia Zharova, Pavel Umrukhin

#### I.M. Sechenov First Moscow State Medical University, Moscow, 119435, Russia

##### **Correspondence:** Pavel Umrukhin – I.M. Sechenov First Moscow State Medical University, Moscow, 119435, Russia

**Introduction:** We have faced a task of creating a new methodology to predict resistance to stress. We assumed that the animal’s resistance to stress may be predicted based on the level of corticosterone before stress. This research was carried out in comparison with the traditional methodology.

**Methods:** A total of 20 male Wistar rats were tested during the study. In accordance with the traditional methodology, before the blood sampling animals were divided into resistant to stress and stress-prone. Their behavior was studied by the open field test. The group of potentially resistant to stress (active type) included 14 animals with the Motor Activity Index below 0.8.

The animals were divided into two groups, according to the median of corticosterone concentration in the plasma (30.4 ng/ml).

The first blood sampling was realized before stress. In two days animals were put in a conflict situation causing emotional stress. After decapitation of animals the second blood sampling were performed. The level of corticosterone in the plasma before and after stress was studied by the enzyme multiplied immunoassay (EAI).

**Results:** Concentration of corticosterone in the first group made 20.6 [12.3;28.9] ng/ml and in the second – 38.9 [33.6;46.9] ng/ml. While studying distribution of corticosterone concentration before stress in the groups traditionally it was cleared up that in the active group concentration were 26.2 [13.2;33.5] ng/ml and in the passive – 36.3[32.7;52.4] ng/ml.

In the process of comparison of these methodologies it turned out that all animals in the active group had low corticosterone concentration and animals in the passive– high concentration (р < 0.05).

After stress the level of corticosterone in the first group amounted to 42.3 [32.6;72.4] ng/ml and in the second group – 39.3 [26.3;51.2] ng/ml. In the traditionally groups it was cleared up that in the active group concentration were 38.4 [29.5;65.9] ng/ml and in the passive – 44.3[31.8;61.5] ng/ml.

Thus, after stress no relation of the corticosterone level before stress was observed in any group (р > 0.05).

**Discussion:** In the process of comparison of these two methodologies we cleared up that distribution of rats by groups before stress does not vary. However, after stress no methodology is able to predict accurately how the organism will react to stress because after stress 30 % of animals had a lower corticosterone level than originally.

## P17: Investigating the strength model of self-regulation (ego depletion) and medical decision making and error in medical students

### Wendy Evans-Uhegbu^1^, Frank Doyle^2^, Hope Kudryashova^1^, Derek Dorris^3^, Anthony Cummins^4^

#### ^1^Department of Medicine, Royal College of Surgeons in Ireland, Dublin, Ireland; ^2^Department of Psychology, Royal College of Surgeons in Ireland, Dublin, Ireland; ^3^School of Psychology, University College Cork, Cork, Ireland; ^4^Department of General Practice, Royal College of Surgeons in Ireland, Dublin, Ireland

##### **Correspondence:** Anthony Cummins – Department of General Practice, Royal College of Surgeons in Ireland, Dublin, Ireland

**Introduction**

Background: Clinicians are faced with a range of complex clinical cases daily and effective clinical decision making is an essential quality they are expected to possess as this reduces the chances of diagnostic errors and in turn improves safe patient care. A significant fraction of diagnostic errors have been accredited to cognitive bias. There is increasing evidence that Ego depletion affects diverse aspects of an individual’s life.

**Objective**

As the dual model process of clinical decision making- Intuitive (System 1) and Analytical (System 2) is an integral part of a clinician’s daily routine, this study (Experiment 2) building on a recent previous study by Kudryshova (Experiment 1) aims to investigate the link between Ego depletion concentration levels i.e. whether Ego depletion will increase the number of errors and time taken to make clinical decisions.

**Methods**

Fifty medical students in third and fourth year were randomly recruited and assigned to either non ego depleted group 1 or ego depleted group 2 to complete two computer tasks depending on what group they were in. the computer task was a 12 minute numerical task and the second was ten clinically oriented multiple choice questions- three of which were designed to tap into System 2 thinking. The number of errors made, time taken and manipulation scores were recorded.

**Results**

Data analysis showed that there was no significant difference in the total number of errors made, manipulation scores and time taken across both groups.

**Discussion**

This study found that ego depletion had no effect on the clinical decision making skills of third and fourth year medical students. There was no significant difference found in the number of errors, manipulation scores and total time taken to make clinical decisions across the two groups. This could have been as a result of our methodology ranging from the two tasks being somewhat similar to tasks not being sufficiently ego depleting.

## P18: Does bladder drainage with intermittent catheterisation preserve kidney function in boys with posterior urethral valves?

### Jemma Doheny-Shanley^1^, Mark Woodward^2^, Wesley Hayes^2^

#### ^1^Department of Medicine, University of Bristol, Bristol, UK; ^2^University Hospital Bristol NHS Foundation trust, University of Bristol, UK

##### **Correspondence:** Wesley Hayes – University Hospital Bristol NHS Foundation trust, University of Bristol, UK

**Introduction**

Posterior urethral valves (PUV) is the commonest congenital urinary tract anomaly associated with progression to end stage renal disease (ESRD) in children, with bladder dysfunction being a major risk factor. The effect of bladder drainage with intermittent catheterisation on the rate of decline in renal function has not previously been assessed.

**Methods**

A retrospective analysis of changes in the rate of decline in renal function and the degree of hydronephrosis following initiation of bladder drainage was undertaken comparing 10 children undergoing drainage to controls in a single centre cohort of 58 patients with PUV.

**Results**

In the cohort, maximal renal pelvis diameter > 25 mm was associated with increased risk of ESRD (p = 0.005). In patients with bladder dysfunction, initiation of bladder drainage was associated with reduction in hydronephrosis (p < 0.001) but no significant reduction in the rate of decline of renal function (p = 0.6).

**Discussion**

In patients with PUV and bladder dysfunction, initiation of catheter bladder drainage was associated with reduction in hydronephrosis. Whilst no reduction in the rate of decline of renal function was observed in this retrospective analysis, a prospective randomised evaluation of the long-term effect of bladder drainage on renal function is merited.

## P19: Investigating the role of Stonin 2, a Clathrin Mediated Endocytosis adaptor protein, in altered hippocampal synaptic transmission characterized in schizophrenia

### Marina Yostos^1^, David Cotter^2^, Melanie Focking^2^

#### ^1^Royal College of Surgeons in Ireland, Dublin, Ireland; ^2^Department of Psychiatry, Royal College of Surgeons in Ireland, Dublin, Ireland

##### **Correspondence:** Melanie Focking – Department of Psychiatry, Royal College of Surgeons in Ireland, Dublin, Ireland

**Introduction:** Clathrin Mediated Endocytosis (CME), a critical neurotransmission mechanism, is implicated in the hippocampus of schizophrenia patients. 1 Stonin 2, an adaptor protein, regulates CME-dependent internalization of Dopamine D2 and N-methyl-D-aspartate receptors responsible for the abnormal dopamine activity associated with the symptoms of schizophrenia 1.

The aim of the study was to investigate Stonin 2 protein expression in the human hippocampus of schizophrenia versus control samples.

**Methods:** Post-mortem matched midhippocampus samples of 20 schizophrenia and 20 control subjects were obtained from the Stanley Medical Research Institute. The dentate gyrus and cornu ammonis were dissected from the human hippocampus cross-sections. We used Western blotting to investigate patterns of differential Stonin 2 expression among both samples and controlled for equal protein loading by using ERK2 to confirm. Ethical approval has been granted by the Royal College of Surgeons Ethics Committee (REC no. 080).

**Results:** No significant differences in Stonin 2 expression were detected among the samples; further investigation is required to explore qualitative protein differences.

**Discussion:** Expanding on the western blot results by performing a qualitative ELISA will allow investigation of native protein folding ensuring that the quantified protein in the schizophrenia samples is not mutated.

## P20: Predicting complications after colon resection

### Samantha Stancu^1^, Florin Iordache^1,2^, Bogdan A. Popescu^1,2^

#### ^1^Carol Davila University of Medicine and Pharmacy, Bucharest, 020022, Romania; ^2^Bucharest Clinical Emergency Hospital, Bucharest, 014461, Romania

##### **Correspondence:** Bogdan A. Popescu – Bucharest Clinical Emergency Hospital, Bucharest, 014461, Romania

**Introduction:** Colon cancer is the third most common cause of cancer death in both genders, worldwide. Surgical resection is the course of treatment offering a cure for colon cancer, however, it is associated with numerous deleterious postoperative complications. The aim of this study was to identify risk factors associated with postoperative complications in patients who underwent colon resection.

**Methods:** Data concerning demographics, preoperative blood test results, intraoperative parameters and postoperative follow-up was collected from the first 50 patients of an ongoing prospective, observational study comprising 29 males and 21 females with ages between 30–92 years and an average age of 67.8 ± 15.2 years. Starting with May 17th, 2015 patients that underwent colon resection procedures ranging from segmental to total colectomy were enrolled in this study. However, patients diagnosed with tumors requiring a Hartmann rectosigmoidectomy were excluded from this research. Descriptive statistical analysis was carried out using GraphPad Prism with statistical significance set at p <0.05.

**Results:** A total of 81 immediate postoperative complications were encountered in 39 patients (78 %), which were classified into local (n = 21, 25.9 %) and general (n = 60, 74.1 %) categories. Multiple complications were present in 22 patients (44 %). Secondary anemia was the most frequently encountered complication in this study, with 18 cases, accounting for 22.2 % of all complications. Re-operation was deemed imperative in 6 cases (12 %) due to subhepatic and parietocolic abscesses as well as peritonitis secondary to anastomotic leakage. An 8 % mortality rate (n = 4) was computed. All of the deceased patients had been re-operated within three days prior to death. Five risk factors associated with complications after colon resection were established as part of this study: age over 60 years (p = 0.01), manual anastomosis (p = 0.0003), smoking (p = 0), thrombocytosis (p = 0.04) and heart rate ≥ 90 beats per minute (p = 0.00001).

**Discussion:** Colon resection, even when limited to segmental colectomy, is an extensive procedure prone to complications. As such, postoperative complications not only jeopardize an uneventful postoperative recovery for patients, but also account for prolonged hospital stay resulting in excessive costs for the healthcare system. Identification of risk factors is the first step needed in order to further elaborate a stratification system for predicting complications after colon resection. Stratifying patients into categories based on their risk for developing postoperative complications could subsequently contribute to a decreased burden of postoperative complications.

## P21: Knowledge, attitude and practice of the methods of primary and secondary prevention of cervical cancer among NYSC members in Lagos state, Nigeria

### Muhammad-Mujtaba A. Akanmu, Alero A. Robert, Ezekiel O. Oridota

#### University of Lagos, Lagos, Nigeria

##### **Correspondence:** Ezekiel O. Oridota A. Akanmu – University of Lagos, Lagos, Nigeria

**Introduction:** Cervical cancer ranks second highest in terms of incidence, prevalence and mortality in females in Nigeria. This is of huge concern as it is preventable by vaccination and treatable if detected early. As young graduates, NYSC members are in a position to take decisions as regards their own health, educate and advise their parents, younger siblings and relations, community members, spouses as well as future children on cervical cancer and its prevention.

Aim: To determine the knowledge, attitude towards, practice and factors affecting the practice of the methods of primary and secondary prevention of cervical cancer among NYSC members.

**Methods:** A cross-sectional self-administered questionnaire based survey was conducted among 348 NYSC members in Lagos state using an adapted questionnaire. Independent variables - socio-demographics. Dependent variables - knowledge, attitude, practice and factors affecting the practice of cervical cancer prevention methods. The systematic random sampling method was used. Data collected was inputted and analysed using the Epi info v7.1.5.0 package. Chi square or Fisher’s exact tests were used to find associations between dependent and independent variables. Significance levels of 0.05 were used.

**Results:** About 73.72 % and 96.79 % had poor knowledge of cervical cancer and its prevention methods respectively. Nearly 70 % of respondents had good attitudes towards cervical cancer. There was high willingness to undergo screening (76.67 %). About 93.91 % had not taken a dose of the HPV vaccine. Majority of females (90.91 %) had never been screened. About half (51.92 %) of respondents were unwilling to vaccinate their future 9–13 year old children. Identified constraints include lack of knowledge on cervical cancer, its prevention methods, locations to access services, fear of side effects and fear of promiscuity in vaccinated children. Religion, ethnicity and marital status were not significantly associated with dependent variables. Individuals whose source of information was the internet were more likely willing to practice cervical cancer prevention methods than those from other sources.

**Conclusion:** Poor knowledge of cervical cancer and its prevention methods was found. There was good attitude towards cervical cancer. Practice of prevention methods was poor. As young graduates, NYSC members are future decision makers both in the family and society at large. Targeting interventions towards this population may be instrumental to the goal of reducing incidence and mortality rates in Nigeria.

## P22: Incidental glucose and lipid metabolisms disorders among office workers: a cross sectional study

### Ahmad A. Mirza, Ali K. Alzahrani, Omar Alfarhan, Essam Nour Eldin

#### Faculty of Medicine, Umm Al-Qura University, Makkah, Saudi Arabia

##### **Correspondence:** Essam Nour Eldin – Faculty of Medicine, Umm Al-Qura University, Makkah, Saudi Arabia

**Introduction:** Glucose and lipid metabolisms disorders, including diabetes mellitus, pre-diabetic syndrome and dyslipidemia, are well-known risk factors of cardiovascular diseases with an increasing incidence worldwide. Sedentary workers are among the subjects most exposed to develop metabolism disorders. So, we aimed to evaluate the glucose and lipid metabolisms disorders in this specific population.

**Methods:** This was a cross-sectional study carried out in a selected public establishment in Makkah city in Saudi Arabia, in which all Saudi male office workers aged 20 and more having no history of diabetes, pre-diabetes, dyslipidemia or any cardiovascular disease were enrolled. After more than 12 hours of fasting, eligible respondents (141, 73.8 %) underwent a clinical and biological examination including body mass index (BMI) calculation, fasting blood glucose (FBG), 2 hours postprandial glucose tolerance test (PGTT) and lipid profile including LDL cholesterol (LDL-C) and HDL cholesterol (HDL-C).

**Results:** The incidence of diabetes (FBG ≥ 126 mg/dl and/or PGTT ≥ 200 mg/dl) was 22.7 %; while the incidence of pre-diabetic syndromes was 32.7 %, distributed as follows: 17 (12.1 %) cases of impaired FBG defined by a FBG 100–125 mg/dl and 29 (20.6 %) cases of impaired PGTT defined by a PGTT 140–199 mg/dl. With regards to lipid metabolism disorders, high LDL-C was found in 29 (20.6 %) cases and low HDL-C was found in 14 (9.9 %) cases. The frequency of pre-diabetic syndrome was highest (31.8 %) in the age category of 41 to 50 years old (p > 0.001) and increased with BMI (p = 0.027); while the frequency of diabetes was highest in the age category older than 50 years (p < 0.001) with no significant correlation with BMI (p = 0.094). Comparison of lipid metabolism profiles between subjects newly diagnosed as diabetic and those with normal glucose metabolism profile showed higher levels of Cholesterol (260.0 mg/dl versus 207.3 mg/dl, p = 0.001), Triglycerides (208.7 mg/dl versus 161.6 mg/dl, p = 0.019) and LDL-C (161.1 mg/dl versus 113.2 mg/dl, p < 0.001) but no significant difference in HDL-C (59.3 mg/dl versus 61.2 mg/dl, p = 0.802), respectively. Similarly, LDL-C was higher in the pre-diabetic group comparing to the healthy group (p < 0.001).

**Discussion:** There is a high incidence of glucose and lipid metabolisms disorders in office workers, indicating a high cardiovascular risk in this population. The atherogenic dyslipidemic profile of the pre-diabetic office workers is similar to that in the diabetic peers. This study exposed the need for implementing systemic screening programs for cardiovascular risk factors as a priority of occupational health in sedentary professional settings.

## P23: Differentiating clinically significant spinal injuries; a review of emergency department presentations

### Bronagh MacManus^1^, Owen Keane^2^, Patrick Hillery^2^, James Lee^2^, Hugh O’Reilly^2^, Niamh Collins^2^

#### ^1^Royal College of Surgeons in Ireland, Dublin, Ireland; ^2^Connolly Hospital Blanchardstown, Royal College of Surgeons in Ireland, Dublin, Ireland

##### **Correspondence:** Niamh Collins – Connolly Hospital Blanchardstown, Royal College of Surgeons in Ireland, Dublin, Ireland

**Introduction:** Spinal immobilisation in trauma patients aims to prevent unnecessary spinal movement and secondary injury to the spinal cord. However, recent debate has identified that immobilisation itself may have negative consequences and cause harm. The incidence and patient characteristics of those with a Clinically Significant Injury (CSI) presenting to a general Emergency Department is unknown; this study was devised to investigate these matters in the context of an undifferentiated trauma population.

**Methods:** A retrospective review was completed of all radiological imaging of cervical, thoracic and lumbar spine in trauma patients over a 6 month period. The incidence of traumatic spinal injury was determined. Via chart review, all patients with a CSI were identified. CSI was defined as any spinal injury that subsequently underwent surgical or brace stabilisation, as per orthopaedic opinion. The pre-hospital characteristics of patients with CSI was collected from chart/EDIS records on an excel database.

**Results:** Spinal imaging was performed for 623 trauma patients over a 6 month period; there was a total of 617 x-rays, 68 CT’s and 17 MRI’s. A total of 23 patients (3.4 %) had a traumatic spinal injury and 12 patients (2 %) had a CSI. All of the patients had identifiable “red flags” at first point of contact with the health service, however only 60 % were immobilised at that time. No harm ensued to those not immobilised.

**Discussion:** In an undifferentiated population, 1 in 30 patients had a spinal injury and 1 in 50 had a clinically significant injury. All patients with CSI had identifiable “red flags” at their first point of contact with the health service (Ambulance or Emergency Department). This study indicates that greater care is required in practice to ensure that those most in need of immobilisation are actually immobilised, and unnecessary immobilisation is reduced.

## P24: Pattern of renal colic occurrence due to urinary stones during Ramadan and other months of the year at King Abdulaziz Medical City, Riyadh, KSA

### Ibrahim Abu saq^1^, Abdullah Al Mufarrih^2^, Muath Jaafari^3^, Abdullah Al Mahayni^3^, Amen Bawazir^3^, Sultan Alkhateeb^4^

#### ^1^Faculty of Medicine, King Khalid University, Abha, Saudi Arabia; ^2^Faculty of Medicine, Al Maarfe Colleges, Riyadh, Saudi Arabia; ^3^Department of Public Health, King Saud bin Abdulaziz University, Riyadh, Saudi Arabia; ^4^Department of Uritolgical Oncology, Kind Abdulaziz Medical City, Riyadh, Saudi Arabia

##### **Correspondence:** Sultan Alkhateeb – Department of Uritolgical Oncology, Kind Abdulaziz Medical City, Riyadh, Saudi Arabia

**Introduction**

Few studies have investigated the frequency of Renal Colic (RC) secondary to urinary stones during Ramadan, and none examined the seasonal variations of Ramadan, where it may fall into summer or winter.

**Methods**

Retrospective cross-sectional study using medical records of 237 patients admitted through emergency room with a diagnosis of RC secondary to urinary stones over randomly selected periods representing Ramadan in winter and summer and other months as a control over a period of 10 years at King Abdulaziz Medical City, Riyadh, Saudi Arabia.

**Results**

Patients fasting in Ramadan are two times more likely to present with a calculus of Ureter as opposed to calculus of Kidney/other, particularly when the holy month of Ramadan falls into the summer. However, there was no significant difference in the frequency of RC secondary to urinary stone between Ramadan and non-Ramadan months. The mean age of patients with kidney/other urinary calculus is significantly higher than age of Ureter calculus patients (P < .001).

**Discussion**

Fasting Ramadan is associated with a higher chance of presenting with ureteric stone especially when Ramadan is in the summer months. Patients with known kidney stone disease should be warranted and recommended to drink plenty of fluids during the nighttime in Ramadan. These findings need to be confirmed in a population-based study.

## P25: Proteomic analysis reveals novel AIB1 co-factors that may contribute to acquired endocrine resistance in breast cancer

### Amenah Dhannoon^1^, Damir Vareslija^2^, Arnold Hill^2^, Leonie Young^2^

#### ^1^Royal College of Surgeons in Ireland, Dublin, Ireland; ^2^Endocrine Oncology Research Group, Department of Surgery, Royal College of Surgeons in Ireland, Dublin, Ireland

##### **Correspondence:** Leonie Young – Endocrine Oncology Research Group, Department of Surgery, Royal College of Surgeons in Ireland, Dublin, Ireland

**Introduction:** Aromatase inhibitor (AI) is the treatment of choice in postmenopausal breast cancer patients and is very successful at improving overall and disease-free survival in patients. However, 30 % of patients do eventually develop resistance leading to disease progression and death. Previous studies have linked this to a steroid receptor co-activator, which is particularly amplified in breast cancer called AIB1. In a Rapid Immunoprecipitation of Endogenous proteins (RIME) study conducted by RCSI breast cancer research group, AIB1 transcriptional co-activators specific to AI resistant cell line model were identified. In this study our objective was to compare the level of these AIB1 coactivators using treatment resistant-cell lines Letrozole resistant (LetR) and responsive cells (MCF-7) using Western Blotting. Our second objective was to confirm the physical interaction between AIB1 and these proteins using Co-Immunoprecipitation (Co-IP) and thereby validate RIME findings.

**Methods:** Four proteins (MTA-2, PRMT-6, EfP and SAF-B) were selected according to their regulatory roles in breast cancer and their levels were measured in the nuclear and cytoplasmic extraction from both cell lines.

**Results:** Higher protein levels of MTA-2, PRMT-6 and EfP were found in the AI resistant cells when compared to the sensitive ones. Furthermore, the interactions between AIB1 and MTA-2, PRMT6 and Efp were validated in the nuclear extracts of AI resistant LetR cells using Co-IP.

**Discussion:** This study has brought us closer to unravelling the AIB1 driven network that potentiates AI resistance. Having this knowledge about the environment in which AIB1 functions in the resistant setting can offer a precious insight for researchers to identify patients who are resistant or may acquire resistance during treatment. This can allow us to potentially target AIB1 and its resistance promoting interaction network as a way of overcoming resistance to AI therapy.

## P26: Improving sedation practice in general ICU in Beaumont Hospital

### Declan Donoghue^1^, Criona Walsh^2^, Aileen McCabe^2^, John Pope^2^, Saturnino Pasco^2^, Caroline Fallon^2^, Don Solanki^2^, Fiona Kiernan^2^, Sinead Galvin^2^, Jquan Mucvimicc^2^, Johanna Mulvihill^2^

#### ^1^Department of Medicine, Royal College of Surgeons in Ireland, Dublin, Ireland; ^2^ICU Department, Beaumont Hospital, Beaumont, Dublin, Ireland

##### **Correspondence:** Johanna Mulvihill – ICU Department, Beaumont Hospital, Beaumont, Dublin, Ireland

**Introduction:** Early deep sedation in the ICU is associated with poorer outcomes. The Richmond agitation sedation scale (RASS) assesses adequacy and depth of sedation. A clinical microsystem is an organising framework that provides a conceptual and practical approach for multidisciplinary teams (MDT) working in discrete units to deliver and improve the quality of patient care. This study aims to utilise elements of a clinical microsystems structure to increase RASS target setting and assessment in the first 48 hours.

**Fig. 1 (abstract P26).**
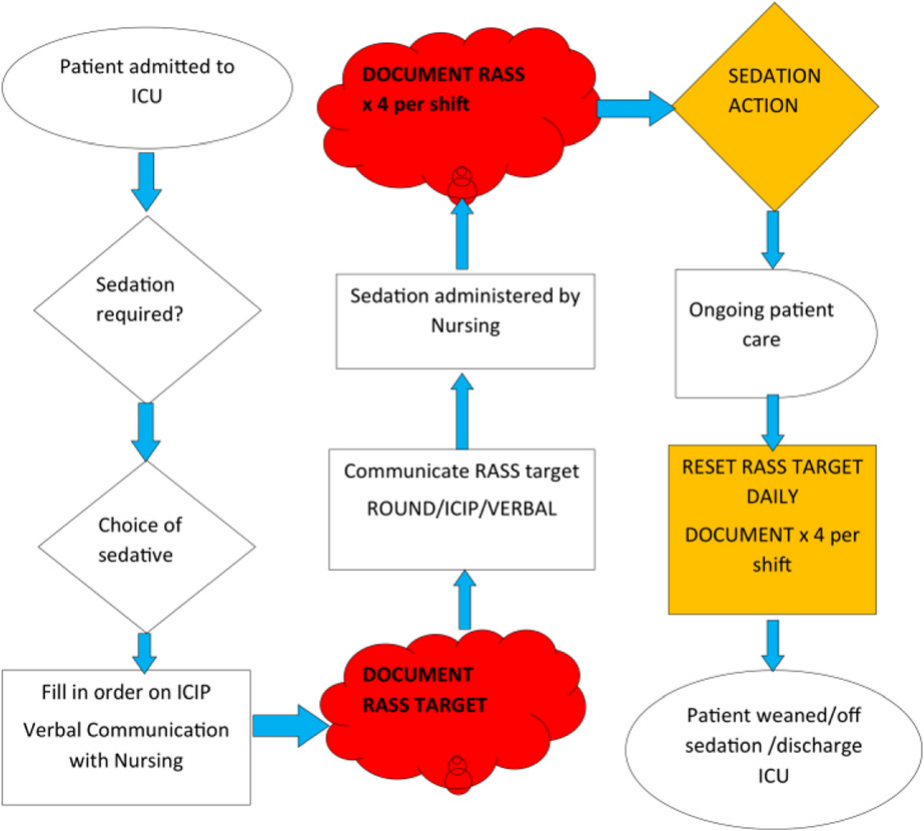
Model on how to implement change in a critical care setting

**Methods:** An observational before-after cohort study of patients admitted to general ICU in August 2014 and August 2015 was undertaken. Adults requiring mechanical ventilation, admitted for at least 12 hours were included; those sedated as part of intracranial pressure (ICP) management protocols were excluded. Following MDT engagement, the process of sedation was mapped as per microsystem methodology (Fig. 1). A fishbone diagram to analyse potential barriers in utilising the RASS effectively was prepared. An educational initiative was planned and delivered to address the weaknesses and barriers identified in the process. Descriptive statistics and appropriate group comparisons analysis was performed.

**Results:** Eighteen out of a possible 39 patients were suitable for analysis in August 2014, and 13 out of a possible 38 were included in August 2015. No significant difference in group demographics was observed. RASS scoring increased significantly in the the first 48 hours of admission from 9.3 ± 4 to 13 ± 5.7 assessments (p = 0.041).

**Discussion:** Increased RASS target setting and assessment was observed after the intervention. The scale of the study is too small to draw definite conclusions, but serves as a model on how to implement change in a critical care setting (Fig. 1).

## P27: Diagnosis and control of hypertension as indicators of the level of awareness among relatives of medical students

### Ahmad A. Mirza^1,2^, Soha A. Elmosry^3,4^

#### ^1^College of Medicine, Taif University, Taif, Saudi Arabia; ^2^Research Center, King Abdullah Medical City, Makkah, Saudi Arabia; ^3^Department of Pharmacology, Faculty of Medicine, Cairo University, Cairo, Egypt; ^4^Epidemiology and Statistics Department, King Abdullah Medical City Research Center, Makkah, Saudi Arabia

##### **Correspondence:** Soha A. Elmosry – Department of Pharmacology, Faculty of Medicine, Cairo University, Cairo, Egypt

**Introduction:** Patients’ awareness plays an important role in the early diagnosis and control of many diseases including hypertension. We aimed to estimate the level of awareness among the relatives of medical students by assessing the prevalence of undiagnosed and uncontrolled hypertension.

**Methods:** In this cross-sectional designed study, a certian group of medical students were invited to interview their respective adult first-degree relatives for risk factors and take measurement of their blood pressure in adequate conditions. According to the absence or presence of hypertension in their history, relatives who were measured with elevated blood pressure were analyzed as undiagnosed or uncontrolled hypertension, respectively; while those who were measured with normal values of blood pressure were analyzed as normal or controlled hypertension, respectively. Comparative analysis of different parameters was carried out between these subgroups.

**Results:** We included 770 relatives of 82 (57.7 %) total students’ participations. The prevalence of undiagnosed hypertension in the total study population was 14.4 % (111 cases). Among participants diagnosed previously with hypertension, 61.9 % were uncontrolled at the time of the study. Predictors for undiagnosed hypertension were aged below 40, working at the present time in either governmental or private sectors, current smoking, absence of diabetes and cardiac diseases; while none of the investigated factors showed to be a significant predictor for uncontrolled hypertension.

**Conclusions:** There is insufficient level of awareness among the family members of medical students, as demonstrated by the high prevalence of both undiagnosed and uncontrolled hypertension. The typical profile associated with the lowest awareness level is that of the young smoking employee with no history of diabetes or cardiac disease.

## P28: Evaluation of the antitumor potential from extracts of endemic plants of Brazilian caatinga against melanoma and hepatocarcinoma

### Lorenza Andres Ameida De Souza, Yuri de Oliveira, Diego Menezes, Alene Vanessa Santos

#### Núcleo de Biotecnologia e Bioprospecção (NBBio), Escola Bahiana de Medicina e Saúde Pública, Salvador, Brazil

##### **Correspondence:** Alene Vanessa Santos – Núcleo de Biotecnologia e Bioprospecção (NBBio), Escola Bahiana de Medicina e Saúde Pública, Salvador, Brazil

**Introduction:** Cancer is responsible for approximately 13 % of all deaths worldwide, because of this it is characterized as a public health problem. There are still many difficulties associated with finding treatment, such as high toxicity of the drugs, not targeting only the tumor cells, water insolubility and increasing refractory cases. Currently, the number of projects developed around bioprospecting involving substances from endemic plant extracts of various biomes has increased significantly, this growth is due to the fact that many of the drugs used in cancer treatment are derived from natural products, for example: Vincristine and Vinblastine (Vinca rosea), Paclitaxel and Docitaxel (Taxus brevifolia), Etoposide and Teniposide (Podophyllum peltatum and P. emodi). In this research our project aimed to evaluate the antitumor potential of extracts from endemic plants of Brazilian Caatinga.

**Methods:** This investigation used two cell lines: HEPG2 (hepatocarcinoma) and B16F10 (murine melanoma), which were grown in plastic bottles using RPMI® medium supplemented with fetal bovine serum and antibiotics and incubated at 5 % CO2. The assessment of proliferation was verified by the methylene blue colorimetric test after the cells were incubated on plates treated with the extracts at a concentration of 100 μg/ml for 24 and 48 hours.

**Results:** The findings of those tests showed that extracts A10F, A11F had a statistically significant of grow inhibition more than 80 % at 24 and 48 hours with B1610 cells. The extract A19F showed the average of growth inhibition higher than 89 % at 24 and 48 hours in both cell lines.

**Discussion:** Thus, expected in the long term is the development of further research to identify: potential cellular targets of action, the type of death and the biochemical pathways involved. After in vitro screening of the extracts we aim to isolate the active substances and conduct in vivo tests.

## P29: The role of Chromogranin A as a biomarker in drug resistant neuroblastoma

### Ahmad Zaki Asraf^1^, Raymond Stallings^2^, Olga Piskareva^3^, Ross Conlon^3^

#### ^1^Department of Medicine, Royal College of Surgeons in Ireland, Dublin, Ireland; ^2^Cancer Genetics Department, Royal College of Surgeons in Ireland, Dublin, Ireland; ^3^Molecular and Cellular Therapeutics Department, Royal College of Surgeons in Ireland, Dublin, Ireland

##### **Correspondence:** Ross Conlon – Molecular and Cellular Therapeutics Department, Royal College of Surgeons in Ireland, Dublin, Ireland

**Introduction:** Neuroblastoma is the commonest solid extracranial paediatric malignancy of sympathetic nervous system, accounting for 15 % childhood cancer mortality despite multimodal treatment. The acquisition of multidrug resistance is a major impediment to the successful treatment of high-risk neuroblastoma, where induction treatment often includes cisplatin in combination with other drugs. Importantly, cisplatin treatment is known to infer cross-resistance to other chemotherapeutic drugs. Chromogranin A (CgA) is a promising biomarker to use as a diagnostic tool as compared to other serum biomarkers. The two main objectives of the project are to investigate CgA as a circulating biomarker in response to chemotherapy in drug resistant neuroblastoma and to demonstrate expression levels of CgA in neuroblastoma tumours sensitive and resistant to chemotherapy.

**Methods:** Examination of CgA expression in tumours by western blotting analysis: 4 parental tumour cells (Kelly, KellyCis83, KellyLuc, and KellyCis83Luc) were run on SDS-PAGE using primary antibody (ab68271) and secondary antibody (ab6721-1). Bands were then detected on autoradiographic films using enhanced chemiluminiscence detection. CgA expression in plasma by ELISA: 33 plasma samples of different cohorts (sensitive tumours untreated, sensitive tumours treated, resistant tumours untreated, and resistant tumours treated) were prepared. CgAnalyze Kit for CHGA (DAKO, K2378) was set up and samples were added into the well plate per protocol and was then analysed under spectrophotometer. Standard curve was plotted to determine relative concentration of CgA in plasma samples. The relationship between tumour resistance and CgA expression was analysed by unpaired t-test. A p-value less than or equal to 0.05 was considered statistically significant.

**Results:** We could not appreciate any differences in CgA expression in drug resistant and drug sensitive neuroblastoma tumour cells in response to chemotherapy due to high backgrounds caused by murine immunoglobulins which is the same size as CgA at 49 kDa. We demonstrated that elevated levels of circulating CgA correlate with tumour’s size regardless of drug resistance status (p < 0.0001) and the levels of circulating CgA are significantly higher in drug resistant tumours versus sensitive in small (<2.00 g) and large (>2.00 g) tumour groups with p < 0.0278 and p < 0.0397 respectively.

**Discussion:** We could not detect any differences in CgA expression in drug resistant and drug sensitive neuroblastoma tumour cells in response to chemotherapy. We demonstrated that elevated levels of circulating CgA correlate with tumour’s size regardless of drug resistance status and the levels of circulating CgA are significantly higher in drug resistant tumours versus sensitive in small and large tumour groups. This data would provide a proof of the concept for circulating CgA as a biomarker for tumour response to chemotherapy in neuroblastoma.

## P30: Membrane sweep at term gestation in CUMH; a case-control study

### Siún Sweeney-Landers^1^, Cathy Burke^2^

#### ^1^Department of Medicine, University College Cork, Cork, Ireland; ^2^General Gynaecology, University Maternity Hospital Cork, Cork, Ireland

##### **Correspondence:** Cathy Burke – General Gynaecology, University Maternity Hospital Cork, Cork, Ireland

**Introduction:** Sweeping the membranes is an obstetric procedure thought to reduce pregnancy duration, by hastening the onset of labour. However, the efficacy of the procedure has been disputed within the literature. Therefore, our study compared total pregnancy duration, interval to labour, and need for formal methods of induction, between study and control groups, in order to determine just how effective the procedure is.

**Methods:** This retrospective case control study examined 250 women, who attended Cork University Hospital from May to July 2015. The study group had been swept whilst the control group had not. SPSS and StatPlus were employed to analyse the data, using t-tests for continuous data and Chi square tests for categorical data.

**Results:** The mean gestation was greater in those who had their membranes swept, 284.02 days (CI 273.25–295.29) vs 281.55 days (CI 273.175–290.27) in those not swept, p-value 0.003. The mean interval to labour was greater in the study group, 5.02 days (CI 4.25–6.29) vs the control group, 4.41 days (CI 4.18–4.99), p-value 0.25. The procedure did not have a significant effect on the need for formal methods of induction with 33 % of those swept being formally induced, whilst 37 % of the control group were formally induced, p-value 0.67.

**Discussion:** Sweeping the membranes is ineffective at reducing overall pregnancy duration and interval to labour. There is a small reduction in the need for formal induction, in those swept, but it is not statistically significant.

## P31: Study of the variability of glucose levels in patients with diabetes undergoing continuous glucose monitoring

### Paraic Behan^1^, Seamus Sreenan^2^

#### ^1^Department of Medicine, Royal College of Surgeons in Ireland, Dublin, Ireland; ^2^Connolly Hospital, Connolly, Dublin, Ireland

##### **Correspondence:** Seamus Sreenan – Connolly Hospital, Connolly, Dublin, Ireland

**Introduction:** Appropriate assessment of glycaemic fluctuation is vital in avoiding complications associated with diabetes mellitus. In this study, the effectiveness of Continuous Glucose Monitoring (CGM) was assessed in comparison to Self Monitoring of Blood Glucose (SMBG) via finger prick testing and HbA1c. We also assessed how well CGM results are incorporated by clinicians into the decision making process.

**Methods:** The glucose levels of 18 patients with T1DM who had undergone CGM were retrospectively analysed. Parameters of hyperglycaemia, hypoglycaemia and glycaemic variability were analysed and compared between the three evaluation methods; SMBG, CGM and HbA1c. Patients’ medical records were also reviewed.

**Results:** Correlations were found between the mean glucose levels generated by CGM and SBGM. HbA1c was seen to correlate strongly with SBMG and in the case of CGM, it correlated with mean and % hyperglycaemia time but not % hypoglycaemia time. Within CGM, there was a poor correlation between measures of glycaemic variability and % hypoglycaemia time and % hyperglycaemia time. CGM brought about a fall, albeit insignificant, in average HbA1c levels. CGM was mentioned in the decision to alter treatment regimens of 73.3 % of the patients studied.

**Discussion:** SBGM and HbA1c are as effective as CGM in detecting periods of hyperglycaemia. CGM is more effective than HbA1c in detecting periods of hypoglycaemia. Measures of glycaemic variability need further analysis to assess their worth. CGM should be reviewed more frequently by the health care team to inform changes in therapeutic regimen.

## P32: Inflammatory cytokine response to decreased plasma alpha-1 antitrypsin levels in individuals with the MZ genotype

### Ahmed Organjee^1^, Tatsiana Crosbie-Staunton^2^, Emer Reeves^2^, Noel McElvaney^2^

#### ^1^Department of Medicine, Royal College of Surgeons in Ireland, Dublin, Ireland; ^2^Education and Research Center, Beaumont Hospital, Beaumont, Dublin, Ireland

##### **Correspondence:** Noel McElvaney – Education and Research Center, Beaumont Hospital, Beaumont, Dublin, Ireland

**Introduction:** In response to inflammation alpha-one antitrypsin (AAT) is produced by the liver to regulate the inflammatory process and to limit lung tissue damage by inhibiting proteolytic enzymes such as neutrophil elastase and production of pro-inflammatory cytokines. The AAT gene is located on chromosome 14 and has variant alleles including M, S and Z. Healthy individuals carry two forms of the normal gene M, while individuals with deficient AAT levels carry one or two forms of the S or Z gene. As a result they are at higher risk of developing chronic obstructive pulmonary disease (COPD) and cirrhosis, and exposure to environmental factors increases their risks of developing disease at a younger age. The project aim was to assess differences between healthy individuals (MM) and individuals with the intermediate deficiency (MZ), with focus on plasma levels of circulating inflammatory mediators.

**Methods:** Ethical approval was obtained from Beaumont Hospital Ethics Committee. Plasma samples (N = 36) were collected from MM and MZ individuals. All participants were categorized according to their genotype and smoking status. A membrane based cytokine array was used to detect the presence of specific cytokines in pooled plasma samples. Enzyme-linked immunosorbent assays (ELISA) for plasma levels of IL-6, IL-8 and IL-17 were used to validate array data.

**Results:** Results obtained from the cytokine array demonstrated differences between MM and MZ individuals in 44 cytokines with regard to their level of expression. Smoking was shown to affect the expression of a range of cytokines in MM and MZ individuals.

Validation of the array results by ELISA revealed that IL-6 and IL-8 (non-smoker) data support results obtained by cytokine arrays with a trend towards decreased levels, however, data were not statistically significant. In contrast however, IL-17 data demonstrated two statistically significant differences between the MM-non-smoker (1.673 ± 0.1489 pg/ml) and MZ-non-smoker groups (1.353 ± 0.05073 pg/ml) (p = 0.0284) and between MM-non-smoker and MM-smoker groups (1.345 ± 0.04980 pg/ml) (p = 0.0380).

**Discussion:** Cytokine analysis supports the involvement of a range of cytokines, chemokines and cell types, in addition to neutrophils, in the pathology of the disease. This can be concluded from the change in cytokine levels in pooled samples of MM and MZ smokers and non-smokers. This potentially demonstrates that individuals with low levels of AAT are more prone to develop COPD or other inflammatory related diseases. We conclude that augmentation therapy may be of benefit in treatment of MZ-individuals with COPD or other manifestations of AAT deficiency.

## P33: Analysing the role of SRC-1 in breast cancer stem cell formation and activity

### Crystal Mieres^1^, Leonie Young^2^, Sara Charmsaz^2^

#### ^1^Department of Medicine, Royal College of Surgeons in Ireland, Dublin, Ireland; ^2^Department of Surgery, Royal College of Surgeons in Ireland, Dublin, Ireland

##### **Correspondence:** Sara Charmsaz – Department of Surgery, Royal College of Surgeons in Ireland, Dublin, Ireland

**Introduction:** Breast cancer stem cells (BCSCs) are a sub-population of cancer cells that exhibit self-renewal, differentiation potential, increased metastatic ability and resistance to therapy [1]. The steroid receptor co-activator-1 (SRC-1) is a transcriptional coregulator which is associated with the promotion of metastatic lesions in breast cancer and resistance to endocrine therapies [2]. These findings have prompted an investigation of the role of SRC-1 in BCSC formation and activity.

**Methods:** In this study, tamoxifen resistant breast cancer cells (LY2) and SRC-1 knockdown cells (LY2ShSRC-1) were compared. Quantitative Polymerase Chain Reaction (QPCR) was used to validate decreased SRC-1 gene expression in the LY2ShSRC-1 cells. CD44+/CD24- BCSC populations were identified using flow cytometry and BCSC activity was assessed using functional assays. Mammopshere and scratch assays were used to analyse the role of SRC-1 in mammosphere formation and cell migration respectively.

**Results:** Results from QPCR analysis demonstrated that SRC-1 expression in the LY2ShSRC-1 cells was decreased by more than 50 %. Using CD44+/CD24- markers, it was revealed that there were less BCSCs in the LY2ShSRC-1 population than in the LY2 cells. Results also highlighted decreased cell migration in the LY2ShSRC-1 cells and a lower capacity to form mammospheres.

**Discussion:** This study suggests that SRC-1 may have an important role in BCSC load and activity. Further studies need to be conducted to investigate the precise mechanisms by which SRC-1 promotes metastasis and therapy resistance.

## P34: Screening Streptococcus pneumoniae isolates for virulence genes

### Aya Al-Jalamdeh^1^, Mary Corcoran^2^, Martha McElligott^2^, Niall Stevens^1^, Hilary Humphreys^3^

#### ^1^Royal College of Surgeons in Ireland, Dublin, Ireland; ^2^Irish Pneumococcal Reference Laboratory, Temple Street Children’s University Hospital, Dublin, Ireland; ^3^Department of Clinical Microbiology, Beaumont Hospital, Royal College of Surgeons in Ireland, Dublin, Ireland

##### **Correspondence:** Hilary Humphreys – Department of Clinical Microbiology, Beaumont Hospital, Royal College of Surgeons in Ireland, Dublin, Ireland

**Introduction:** Streptococcus pneumoniae is a bacterial pathogen that can lead to invasive pneumococcal disease (IPD) and is a leading cause of mortality worldwide. Although over 90 serotypes exist, the pneumococcal conjugate vaccines (PCV7/13) target the predominant serotypes. The aim of this study was to screen a collection of isolates for virulence genes associated with invasive or non-invasive infections and to determine if there is a relationship between these and particular serotypes.

**Methods:** A total of 192 IPD isolates were screened for the presence of eight virulence genes. Isolates were cultured on blood agar and DNA was extracted using the QIAamp kit. Polymerase Chain Reactions were used to detect the eight virulence genes. The results were compared to a collection of strains from non-invasive infections.

**Results:** NanA and nanB were highly distributed in both the invasive and non-invasive infections (>90 %). Most serotypes covered in PCV13 contained more virulence genes compared to non-vaccine associated serotypes. The psrP, rlrA, pclA,sipA, nanC and zmpC genes were absent in the majority of the non-vaccine serotypes. However, some non-vaccine serotypes were found to have more virulence genes including serotype 6C, 10A, 11A, 15A and 15B.

**Discussion:** The high distribution of neuraminidase genes (nanA/B) may provide a selective advantage to pneumococci over other commensal organisms. Certain serotypes contained a number of virulence genes. This should be considered during vaccine development in order to target the more virulent serotypes and reduce the burden of disease.

## P35: Assessment of the relevance of admission clerking criteria taught to medical students at King Abdulaziz University to real hospital practice

### Rashid Barnawi, Abdulaziz Ghurab, Sultan Alfaer, Hassan Balubaid, Kamal Hanbazazah, Mohammed Bukhari

#### Faculty of Medicine, King Abdulaziz University, Jeddah, Saudi Arabia

##### **Correspondence:** Mohammed Bukhari – Faculty of Medicine, King Abdulaziz University, Jeddah, Saudi Arabia

**Introduction:** Medical record is an important tool for patient care in hospital setting. An essential part of a medical record is the history taking and physical examination. At King Abdulaziz University (KAU), medical students are taught to take a detailed history and perform a thorough physical examination to all patients at the time of their admission. Interns and residents who do the admission clerking are therefore expected to have a similar approach. We are questioning the relevance of what medical students are taught to do to what intern/residents actually do in real practice as well as the completeness of their admission clerking notes.

**Methods:** Retrospective study of 860 admission notes of patients who were admitted for the first time during two months from 1st of December 2014 to 31st of January 2015 to seven departments. Admission notes were reviewed through the electronic medical record health information system and were evaluated using a checklist structured using the referenced textbooks for medical students. Elements of the history taking and physical examination in the checklist could be evaluated as informative, less informative, non-informative, not present or N/A. Data obtained was analyzed using SPSS version- 23 and was summarized into frequency and relative frequency. Using Guilbert’s equation, Average informativeness index was calculated to investigate the extent of informativeness of the notes collectively and for each department.

**Results:** The overall average informativeness index was 50 %, which is unsatisfactory. For Paediatrics admission notes, the average informativeness index was 54 %, followed by Gynecology admission notes 53 %, ICU 52 %, Isolation unit 51 %, CCU and Surgery 50 % and finally Medicine 49 %. In the history taking part of the checklist, notes of “associated symptoms” were the most informative (82.4 %) while previous episodes, family history medication and allergies were informative in 5.6 %, 2.9 %, 4.8 % and 1.9 % of the notes, respectively. The physical examination part shows an overall extreme lack of informativeness, especially in CNS, abdomen, respiratory and heart examination, as they were informative in 4.2 %, .9 %, .7 % and .2 % of the notes, respectively. Breast examination was not documented informatively in any of the notes.

**Discussion:** Results of this study delineate that interns and residents do not completely follow the standards that are taught to medical students and their notes are incomplete, which will affect the integrity of the medical record and adherence of medical students to these standards. Focus group discussion should be conducted regarding the reasons as an attempt to solve this issue.

## P36: Pattern of emergency department visits during Hajj period

### Mohammed Alsakkaf^1,2^, Ahmad Mirza^2,3^, Amrallah Mohammed^4^

#### ^1^Department of Medicine, Batterjee Medical College, Jeddah, Saudi Arabia; ^2^Research Center, King Abdullah Medical City, Makkah, Saudi Arabia; ^3^College of Medicine, Taif University, Taif, Saudi Arabia; ^4^Oncology Department, King Abdullah Medical City, Mekkah, Saudi Arabia

##### **Correspondence:** Amrallah Mohammed – Oncology Department, King Abdullah Medical City, Mekkah, Saudi Arabia

**Introduction:** Hajj is a religious duty performed by Muslims at least once in their lifetime. Approximately over two million people from different parts of the world. They are usually elderly, performing the same rituals by everyone over a short period of time, resulting in over-crowding so increase the risks and complications. During hajj the hospitals in holy areas face a huge change in pattern of emergency visits.The aim of this work is to describe the pattern of the emergency department visits (EDVs) among hajj patients and to explore their causes and potential avoidability of unneeded visits

**Methods:** The study was conducted at King Abdullah Medical City, TCH in Makkah, Saudi Arabia. The study included all Hajj patients admitted during the period from1 Sept. to 13 Oct.2015. Demographic, disease features and registered timeline were obtained. The EDVs that ended by discharge were considered avoidable visits.

**Results:** A total of 199 charts of Hajj patients were reviewed, of whom 117 were male, in total 232 EDVs were analyzed. 8.6 % patients referred from other healthcare centers, of which (2.5 %) patients were transferred from Mashair hospitals (Mina, Arafat hospitals).The most common diagnosis was related to cardiovascular diseases (33.7 %). However, trauma cases represented only 7.8 %. Most of the patients visited ED once (87.4 %), however, some sought ED more than one time (12.6 %). The major visits were encountered during shift A (08:00–15:59, 48 %) followed by shift B (16:00–23:59) and C (00:00–08:00), 42 % and 10 %, respectively. The EDVs occurred in shift A (08:00 to 16:00 h), in shift B (16:00 to 00:00 h) and in shift C (00:00 to 00:08 h) were 48 %, 42 % and 10 %, respectively, extended for an average of 3 ± 2.9 hours, and ended by hospitalization in 55.6 %. Of 103 discharged patients, 61.6 % visited during shift A. The admission occurred mostly in shift A and B and the difference was remarkably significant (P < 0 .001). However there was no significant difference in time spent in ER between EDVs that ended by hospitalization compared with those discharged. Most of patients presented in ER consider in CTAS level 3 (50.5 %) then CTAS level 4 (30.9 %) While in CATAS level 1 only 1 %.

**Discussion:** A significant proportion of EDVs is potentially avoidable. A Comparison in the ED between hajj and non-hajj periods is necessarily to expose different factors disclosed confined health services, which might be present in hajj.

## P37: Anti-Dengue activity of Aspergillus terreus (sulochrin); An in vitro study

### Anastasia Pratanata^1^, Maria Nathania^1^, Tsabita Annisa^1^, Beti Dewi^2^

#### ^1^Faculty of Medicine, Universitas Indonesia, Jakarta, Indonesia; ^2^Department of Microbiology, Faculty of Medicine, Universitas Indonesia, Jakarta, Indonesia

##### **Correspondence:** Beti Dewi – Department of Microbiology, Faculty of Medicine, Universitas Indonesia, Jakarta, Indonesia

**Introduction:** To date, neither vaccine nor anti-viral has been approved to address dengue fever. Considering its annual occurrence of more than 150000 cases, dengue fever poses a substantial burden to Indonesian public health sector; thus making discovery on therapeutic agent against DENV imperative. Previous study indicates a potent Anti-Hepatitis C virus (HCV) activity of Sulochrin, a compound isolated from Aspergillus terreus. As DENV and HCV come from the same family of flaviviridae, it is hypothesized that sulochrin might exert similar inhibitory activity. This research aims to investigate the possibility of anti-dengue activity of sulochrin.

**Methods:** To determine half maximal inhibitory concentration (IC50), Sulochrin were administered in various concentrations (80, 40, 20, 10, 5, and 2.5 μg/ml) to DENV2-infected Huh7 cells. Inhibition activity was observed by means of Focus Assay. Toxicity effect was measured through MTT Assay; to determine half maximal cytotoxic concentration (CC50). Selectivity Index was then calculated through ratio of CC50/IC50. To substantiate findings, unpaired T-Test was performed on each treatment, in comparison with Dimethyl Sulfoxide (DMSO) as control.

**Results:** Significant inhibition of 91,78 % (p = 0.005) and 39.73 % (p = 0.02) was observed upon treatment with 80 μg/ml and 40 μg/ml of sulochrin respectively. At concentration 20 μg/ml, growth was inhibited by 17.69 % (p = 0.09). At 10 μg/ml however, there was no inhibition activity demonstrated (p = 0.98). Interestingly, negative value of inhibition was observed upon treatment concentration below 10 μg/ml. At 5 μg/ml and 2.5 μg/ml, there were an improved in replication as much as −11.32 % (p = 0.21) and −2.35 % (p = 0.38). Further, the results also indicate IC50 of 56.86 μg/ml, CC50 of 150.85 μg/ml and Selectivity Index of 2.65.

**Discussion:** Aspergillus terreus (sulochrin) exert both inhibition and enhancement activity towards Dengue virus in vitro. Its well-tolerated cytotoxic activity reflects an advantageous feature for developing anti-viral. In the future, further derivate analysis could be done to find pure compound’s inhibition in order to maximize drug’s potency.

## P38: The comorbidome in alpha-1 antitrypsin deficiency

### Kuok Zhen Lee^1^, Tomas P. Carroll^2^, Laura Fee^2^, Noel G. McElvaney^2^

#### ^1^Royal College of Surgeons in Ireland, Dublin, Ireland; ^2^Alpha One Foundation, Royal College of Surgeons in Ireland, Beaumont Hospital, Dublin, Ireland

##### **Correspondence:** Noel G. McElvaney – Alpha One Foundation, Royal College of Surgeons in Ireland, Beaumont Hospital, Dublin, Ireland

**Introduction:** AAT deficiency (AATD) results from mutations in the AAT gene, classically presenting with chronic obstructive pulmonary disease (COPD) and liver disease. The most common disease-causing mutation is Z (Glu342Lys), with the milder S (Glu264Val) also associated with lung disease. In Ireland, 1 in 25 people are heterozygous for the most common harmful Z mutation.1 AATD is under-diagnosed and prolonged delays in diagnosis are common. ATS/ERS guidelines advocate screening all COPD, refractory asthma, and cryptogenic liver disease cases, as well as relatives of AATD individuals. The commonest manifestations of AATD include pulmonary emphysema, bronchiectasis, liver cirrhosis and hepatic steatosis but conditions such as panniculitis and vasculitis have also been found recently to be associated with AATD. In this study, we explore the different comorbidities among 154 severe AATD patients enrolled in the National AATD Registry.

**Methods:** 153 severe AATD (AAT level <0.6 g/L) patients have been selected from the National AATD Registry. Among these patients, 149 are Z/Zs patients and 4 carry extremely rare mutations including Z/Zbristol, Z/Mmalton, Mmalton/Mmalton and Nullbolton/Nullbolton. A review of each patient’s medical and electronic record was performed to determine their comorbidities. In addition, we looked specifically for evidence of panniculitis and vasculitis among this cohort.

**Results:** We identified a large number of severe AATD individuals with comorbidities (136/153, 88.89 %). Proven comorbidities: The study started off with investigation of PFT results to find out the number of patients with obstructive pulmonary disease with FEV1% predicted as the determinant. 92 patients were found to have FEV1 (% predicted) of less than 80 %. Next, we examined HRCT reports of 154 patient, 94 patients had notable emphysematous changes in their lungs while 74 suffered from bronchiectasis. Overall, there are 20 patients who are free of any pulmonary manifestations and have FEV1 scores > 80 %. Abdominal ultrasound results, on the other hand, revealed that 25 patients showed evidence of hepatic steatosis and 6 other patients have been diagnosed with liver cirrhosis. This study also identified 9 patients with suspected panniculitis, 5 were later confirmed. Regarding the vasculitis association, only 20 patients (13.07 %) in the entire cohort have been screened for this condition. 6/20 had a positive auto-immune results. Comorbidities unrelated to AATD: We proceeded to investigate comorbidities presumed unrelated to AATD. Our results revealed a wide range of conditions as shown in the attached table.

**Discussion:** Our findings demonstrate that AATD individuals often experience a variety of comorbidities. These are either directly or indirectly linked to their primary deficiency or presumed unrelated. Knowledge of comorbidities is vital for the prognosis of each patient and hence should be rigorously identified and closely monitored.

## P39: MLO-Y4 cells behave more like osteocytes in response to mechanical stimulation when cultured in 3D

### Rachel C. White, Robert T Brady, Fergal O’Brien

#### Tissue Engineering Research Group (TERG), Royal College of Surgeons in Ireland, Dublin, Ireland

##### **Correspondence:** Fergal O’Brien – Tissue Engineering Research Group (TERG), Royal College of Surgeons in Ireland, Dublin, Ireland

**Background:** Osteocytes are believed to be the main mechanosensory cell in bone playing an essential role in maintaining bone homeostasis. Fluid shear stress (FSS) stimulates primary osteocytes to promote anabolic processes within bone but little is known about how substrate stiffness (SS) influences osteocyte behaviour. Studies are limited by the inherent difficulty of obtaining and culturing osteocytes in vitro - primary ostocytes are harvested from bone via enzymatic degradation that yields terminally differentiated and damaged osteocytes. The osteocyte-like MLO-Y4 cell line is widely used, however studies report limitations in the production of important osteocyte specific proteins such as sclerostin [2]; a negative regulator of bone anabolism and therapeutic target. Until now, MLO-Y4 studies have only been conducted using 2D substrates, but strikingly these cells are capable of expressing the gene for sclerostin (Sost) when cultured in a 3D scaffold. Using this model, we hypothesised that, since sclerostin has an anti-anabolic influence, it’s gene expression would be greater with increased SS and low in the presence of FSS.

**Methods:** MLO-Y4 cells were seeded onto 3D Collagen-Glycosaminoglycan scaffolds of varying stiffness (0.5kPa, 1.0kPa and 1.5kPa) followed by culture in a static or FSS environment before Sost expression was measured using RT-PCR.

**Results:** There was a linear relationship between Sost expression and increasing SS in the static environment. Across all SS’s, expression of Sost was low when cells were exposed to FSS. The difference in Sost expression was largest between groups 1.5kPa static vs 1.5kPa Flow.

**Conclusions:** Osteocytes interpret greater SS as increased structural integrity evidenced by the increased expression of the anti-anabolic gene Sost. Interestingly, this response is abolished in the presence of FSS indicating that FSS is the dominant mechanical stimulus. Clinically, this is evidence to support exercise/load bearing as a powerful influencer of bone anabolism.

